# Automatability and validity of methods for the quantification of intra-/Intermuscular adipose tissue in conventional MRI: a systematic review

**DOI:** 10.1186/s12880-025-02037-w

**Published:** 2025-11-21

**Authors:** Alicia Pirwass, Birte Glimm, Michael Munz, Hans-Joachim Wilke

**Affiliations:** 1 Institute of Orthopaedic Research and Biomechanics, Centre for Trauma Research, University Hospital Ulm, Helmholtzstrasse 14, 89081 Ulm, Germany; 2 Institute of Artificial Intelligence, Ulm University, 89081 Ulm, Germany; 3 AI for Sensor Data Analytics Research Group, Ulm University of Applied Sciences, 89081 Ulm, Germany

**Keywords:** Intermuscular adipose tissue, Intramuscular adipose tissue, Fatty infiltration, Magnetic resonance imaging, Muscle, Systematic review, Quantitative analysis

## Abstract

**Background:**

Intermuscular adipose tissue (IMAT) and Intramuscular fat (IMF) in skeletal muscle are critical biomarkers associated with functional decline in various musculoskeletal disorders. Routine clinical magnetic resonance imaging (MRI) sequences are frequently employed to quantify IMAT/IMF due to their broad availability and non-invasive nature. However, methodological standardization and comprehensive validation against quantitative MRI (qMRI) reference standards remain sparse. The lack of standardization and validation presents a significant barrier to clinical adoption. Furthermore, automation of IMAT/IMF quantification methods remains underexplored, which limits reproducibility and large-scale application in clinical settings. Addressing these gaps, which is the objective of this systematic review, is essential for ensuring seamless integration of IMAT/IMF quantification into clinical routine assessments.

**Methods:**

Following the PRISMA (Preferred Reporting Items for Systematic Reviews and Meta-Analyses) guidelines, we systematically reviewed 65 studies that assessed IMAT or IMF using conventional MRI. The selected studies were categorized based on their methodological approaches, anatomical regions analyzed, and validation against qMRI reference standards. Additionally, we classified the level of automation of these methods and identified the necessary steps to be implemented in order to reach full automation.

**Results:**

Our findings reveal a high methodological diversity in the literature, with substantial variations based on the anatomical region studied. Very few studies validated their findings against qMRI reference standards, a crucial step for establishing these methods in clinical practice. The automation potential of the reviewed methods varied significantly, with only a limited number of studies addressing full automation.

**Conclusion:**

This systematic review highlights gaps in validation and automation of IMAT/IMF quantification methods using conventional MRI sequences. We provide guidance for researchers and clinicians aiming to implement these techniques in routine assessments. Transitioning from qualitative to quantitative MRI assessments requires standardization and automation to improve reproducibility and clinical applicability. Automation plays a key role in integrating these methods into clinical workflows, reducing manual effort, and increasing efficiency. By fostering the development of computer-aided solutions, this review supports the advancement of reliable and accessible IMAT/IMF quantification methods that have the potential to transform musculoskeletal imaging and patient care.

## Background

Fatty infiltration (FI) has emerged as an important biomarker for muscle pathologies, age-related muscle deterioration, and conditions such as sarcopenia and cachexia [1]. Characterized by the gradual degenerative accumulation of fat within skeletal muscle, FI reflects a pathological process associated with declining muscle quality and function over time. This progressive infiltration can be quantitatively assessed by measuring (IMF), offering a means to track the trajectory of muscular degeneration. Although a universally accepted definition is still lacking, in this study we distinguish clearly between Intermuscular adipose tissue (IMAT) and IMF. IMAT refers to adipose tissue located between individual muscles but outside their respective fasciae, corresponding to perimuscular fat deposits as described by Engelke et al. [1]. In contrast, IMF designates fat within the muscle fascia, encompassing both intramyocellular lipids (IMCL) and extramyocellular lipids (EMCL). This distinction aligns with emerging anatomical consensus and facilitates consistent terminology across studies and anatomical regions. IMF accumulation is of particular relevance in chronic conditions such as back pain and neuromuscular disorders, as it contributes to both disease progression and loss of function [2]. Conversely, in other anatomical regions, attention has been directed toward the quantification of IMAT. Quantifying IMF and IMAT provides essential insights into muscle health and functional reserve, supporting early detection, longitudinal monitoring, and clinical decision-making [3].

Advanced quantitative magnetic resonance imaging (qMRI) techniques, including magnetic resonance spectroscopy (MRS) and chemical shift encoded magnetic resonance imaging (CSE-MRI), are currently considered reference standards for accurately quantifying IMF and IMAT [1, 3]. However, the specialized infrastructure and technical expertise required for these methods often restrict their use in clinical practice, where also the use of reference standard imaging may not be expedient [1]. Consequently, there is a demand for accessible methods that can be seamlessly integrated into routine clinical workflows without requiring additional specialized imaging.

Conventional magnetic resonance imaging (MRI), herein designated as standard clinical fast spin echo sequences with either T1- or T2-weighting, is a well-established modality in clinical imaging. Images of T1-weighted sequences are known to depict fluids as dark, a property that is particularly advantageous for highlighting anatomical structures. On the other hand, T2-weighted sequences highlight fluids as light, making them a go-to method for detecting abnormalities such as edema or inflammation. Conventional MRI sequences are not recommended for the quantification of IMF and IMAT due to the variability of intensity values across sequences and manufacturers [1]. Nevertheless, the utilization of these sequences for the purpose of quantifying IMF and IMAT has seen a notable increase in the existing literature, despite the availability of established qMRI methods. The accessibility of these sequences, in contrast to the more specialized qMRI sequences, might explain the popularity of conventional MRI, which offers the possibility to quantitatively use retrospective data despite its drawbacks. This approach has substantial translational potential, as it could enable the widespread application of IMF and IMAT quantification in clinical practice, improving early detection and monitoring of muscle-related diseases. Methods applied to conventional MRI images to quantify IMF and IMAT vary remarkably in the existing literature, creating an urgent demand for a comprehensive overview of the applied methods, with particular emphasis on their accuracy in contrast to established qMRI techniques. Reliable, validated methods are essential for consistent and accurate quantification across studies [3].

The quantification of IMF and IMAT using conventional MRI has advanced considerably in recent years, driven by a growing demand for standardized and automated analysis tools. By minimizing human intervention, automated approaches offer the potential to enhance reproducibility, enable consistent assessments across sites, and support large-scale studies of muscle degeneration. As such, automation represents a key enabler for bridging the gap between research methods and clinical application.

To support this development, the present systematic review does not aim to compare numerical IMF and IMAT values across studies, but instead provides a structured synthesis of the methodological strategies employed to quantify IMF and IMAT in skeletal muscle using conventional MRI. Specifically, we aim to (i) provide a structured overview of the methodological approaches used for IMF and IMAT quantification in conventional MRI, (ii) their degree of automation using a harmonized classification scheme, and (iii) whether and how the approaches were validated against established reference standards such as Dixon imaging or MRS. By highlighting both the validation status and automation level of each approach, this review offers guidance for selecting appropriate methods and advancing clinically applicable, reproducible workflows.

## Methods

This systematic review is conducted following the guideline of the Preferred Reporting Items for Systematic reviews and Meta-Analyses (PRISMA) 2020 statement [4, 5].

### Eligibility criteria and study design

The systematic review’s eligibility criteria are chosen with the objective of addressing the following guiding question, which follows the PICO (Population, Intervention, Comparison, Outcome) strategy [6]:

Which image analysis methods *(Intervention)* used to quantify the amount of IMF or IMAT in skeletal muscle *(Outcome)* in conventional MRI of humans or animals *(Population)* should be recommended based on automatability and validity *(Comparison)*?

While PICO was originally designed for clinical intervention studies, we adopted it here as a guiding framework due to its clarity and structure, despite slight deviations from its conventional application. Based on this guiding question, an overview of the inclusion and exclusion criteria is displayed in Table 1. With regard to the population, studies that employ imaging methods other than conventional MRI and studies that involve images of other tissue than skeletal muscle are excluded, as this review aims to identify the methodology applied to conventional MRI images of skeletal muscle. The exclusion criteria for the aforementioned studies do not take into account the sex, age, or underlying pathology of the subjects, whether they are human or animal subjects. With regard to the intervention, studies employing any computational image analysis method, such as thresholding, clustering, texture analysis, or deep learning, applied to conventional MRI data were included. Studies were excluded if they did not report the image analysis method used. For the Comparison element of the PICO framework, which we interpret as the assessment of automatability for the comparison of the eligible studies, the same inclusion and exclusion criteria as defined for the Intervention apply. Specifically, studies were only considered if they reported the image analysis method used. The explicit naming of the algorithm or software was deemed sufficient to infer the degree of user interaction and thus the level of automation. Where methodological details were vague or the authors expressed uncertainty (e.g., referring to external tools without specification), this was noted and taken into account in the subsequent evaluation. We excluded studies that estimated IMF and IMAT using visual grading or scoring systems without extracting continuous image-derived metrics. Moreover, studies focusing on the quantitative assessment of biomarkers other than IMF and IMAT in MRI images of muscle are excluded. In addition to the inclusion and exclusion criteria mentioned above, which are based on the guiding question, only original research articles, proposing or evaluating image analysis methods based on retrospective or prospective data, published in peer-reviewed journals or conference papers, are included. Consequently, literature reviews, letters to editors, editorials, and conference abstracts are excluded.

**Table 1:** Inclusion/Exclusion criteria derived from the guiding question according to the PICO framework [6]

Guiding Question	Which image analysis methods (Intervention) used to quantify the amount of IMF or IMAT in skeletal muscle (Outcome) in conventional MRI of humans or animals (Population) should be recommended based on automatability and validity (Comparison)?	
PICO	Inclusion	Exclusion
Population	• Any human or animal of any age, sex or with any pathology receiving conventional MRI of their skeletal muscle	• Other imaging methods than conventional MRI • Images not showing skeletal muscle tissue
Intervention	• Any computational image analysis method (e.g., thresholding, clustering, deep learning) applied to conventional MRI of skeletal muscle	• Studies that do not report the image analysis method used.
Comparison	–	–
Outcome	• Quantitative image-derived measures of IMF or IMAT	• Quantitative assessment of other biomarkers than IMF or IMAT • Qualitative description or semi-quantitative scoring of IMF or IMAT

### Sources of information and search strategy

An in-depth electronic database search was conducted including IEEE explore, PubMed, ACM guide to computing literature, and Web of Science from January 01, 2014 until December 05, 2024. The search strategy employs a comprehensive array of keywords and synonyms extracted from pertinent literature, along with Boolean operators to minimize the likelihood of overlooking relevant studies. Table 2 presents the search string, structured according to the PICO framework, which was applied uniformly across all four databases. In addition to supplementary searches on Google Scholar, the references of the included articles were manually screened in order to identify any further articles that meet the aforementioned eligibility criteria.

**Table 2:** The search strategy developed for IEEE explore, PubMed, acm Guide to computing literature, and web of science. This search string was consistently applied across all databases without any modifications

PICO	Search Strategy
Population	(T2-weighted OR “T1 weighted” OR “T2 weighted” OR T1-weighted OR “T 1-weighted” OR “T 2-weighted” OR T1-MRI OR T2-MRI OR T1w OR T2w)AND (muscul* OR muscle*)
Intervention	–
Comparison	–
Outcome	(”intermuscular fat” OR “intramuscular fat” OR “intramuscular adipose tissue” OR “inter muscular adipose tissue” OR fatty OR infiltration OR morphology OR composition OR degeneration OR atrophy OR “fat content” OR “muscle-fat index” OR “degenerative changes” OR “fat signal fraction” OR “fat fraction”)

### Study selection

After bibliographic research, all references were exported to Zotero 6.0.37 (Corporation for Digital Scholarship, VA, USA), where duplicates were removed electronically, followed by manual removal of the remaining redundant references. Titles were screened first followed by the abstracts, eliminating studies that did not meet the guiding question at each step. For studies rejected during full-text review, the reasons for exclusion were noted. Any unclear decision during the selection process was resolved by consensus among the authors.

### Evaluation of validity and automation

An evaluation of the included studies is conducted with respect to the validity and automatability of the approach used for the quantification of IMF and IMAT.

Validity in this context refers to the accuracy of conventional MRI-based methods for quantifying IMF or IMAT, benchmarked against established reference standard techniques such as MRS and CSE-MRI-based methods such as Dixon, mDixon or IDEAL. Only studies that compare their results with those produced by reference standards for quantifying IMF and IMAT are included in this assessment, as this direct comparison allows the evaluation of how closely conventional methods align with validated techniques in the same patient dataset. Engelke et al. [1] define validity in this context as the method’s ability to reliably and consistently capture true IMF or IMAT across different muscle groups and degrees of infiltration. By focusing on studies that provide this comparison, we can determine each method’s validity rigorously in terms of its concordance with established standards.

Automation describes the extent to which a method can function independently of human intervention, and this review assesses automation levels for each included study. We adapt the definition from Langenbach and Rabe [7] and introduce a level 4* category specific to this review’s context.

The adapted automation levels are as follows:

#### Level 1

Human input and evaluation are required at every stage. Though computer assistance may be used, each step is guided and confirmed by a human operator. For example, pipelines using medical image processing software in which the user manually adjusts a threshold and evaluates the result for each image fall into this category. The concept and application of thresholding are further explained in the Results section.

#### Level 2

Individual processing steps require user confirmation or are completed manually if automation is not possible, although the overall procedure is more automated than in level 1. Typically, users interact with a graphical user interface. For instance, pipelines that calculate a threshold based on the signal intensity of a manually delineated region of interest (ROI) are classified as level 2.

#### Level 3

Similar to level 2, but human involvement is limited to adjustments rather than confirmations, and the user makes the final decision. Likewise, users interact with a graphical user interface, while a fixed threshold, determined from prior knowledge, is applied without the need for manual delineation of a ROI.

#### Level 4*

Complete automation for processing a single image, but with uncertainty about the setup’s capacity for batch processing. For example medical image processing software or source code, that automatically determine a threshold based solely on the signal intensities of an image using any sort of algorithm, but do not specify whether multiple images can be processed consecutively.

#### Level 4

Full automation, encompassing processing and evaluation without human intervention. In contrast to level 4*, methods in this category explicitly support batch-processing. This level enables large-scale, retrospective analysis by processing inputs and presenting results directly.

The additional category level 4* is intended to address the uncertainty regarding full automation, particularly in cases where batch processing capability cannot be determined from the information available in the study. In this context, batch processing is defined as the quantification of IMF and IMAT of multiple images without the need for human intervention. This novel category is particularly aimed at readers considering to adopt any of the tools or quantification methods presented in this review for their own research. It is important to note that, in the absence of established batch processing capabilities, even fully automatic IMF and IMAT quantification for individual images can become a time-intensive task when dealing with large datasets. Moreover, implementing batch processing to handle multiple images efficiently requires prior programming knowledge, which may present an additional barrier for some researchers.

This systematic review aims to identify quantification methods that are both transparent regarding their accuracy and which show promising potential for automated implementation.

### Data collection

The data collection process focuses on systematically retrieving key information from the eligible studies, ensuring a comprehensive overview of each method for quantifying IMF and IMAT in skeletal muscle. The extracted information is the following:

#### Basic study information

Name of the first and last (in this field traditionally the supervisor) author, the country of the affiliations of the last author, title of the study, and year of publication.

#### Anatomical context

While reviewing the eligible studies, notable trends regarding methodology, MRI modality, and segmentation approaches were observed. Consequently, the specific body regions, the exact muscles analyzed in each study and the patients species (e.g., human or animal) were extracted for further evaluation.

#### Cohort details and MRI parameters

Details about the cohort size, MRI weighting, magnetic field strength and other mentioned MRI protocol parameters were recorded to provide context for the imaging environment. Among these parameters, only the MRI weighting is further analyzed in this review, as including additional aspects would have exceeded the scope of the analysis. Nevertheless, the detailed information, particularly the exact parameters used for acquiring conventional MRI images, is undoubtedly valuable for future studies.

#### Preprocessing and quantification methods

Where applicable, the preprocessing algorithm applied to MRI images was noted, but not further analyzed in this review. The segmentation method used for outlining the muscles of interest was extracted. The main method used to quantify IMF and IMAT on conventional MRI images was noted and further categorized based on the underlying principle. Additionally, it was recorded whether a study assessed IMF, IMAT, both in combination, or if it remained unclear which of the two was evaluated.

#### Software and programming language

The software or programming language used for quantification was documented to evaluate the automation potential of each method. Additionally, information was collected on whether the software used is freely available or commercially licensed, as well as on the availability of custom-written code.

#### Validation

If the study included a comparison to a reference standard method, this information was recorded, along with the statistical method used for assessing agreement of the proposed method and the reference standard.

#### Automation level

Each study’s method was assessed for its level of automation as outlined in the previous subsection.

#### Automation requirements

Where applicable, additional steps required for each method to achieve full automation (level 4) were noted.

To ensure consistency and reduce subjectivity in data extraction, a structured data extraction template was developed prior to the review. The template included clear and detailed instructions for each field, specifying the exact information to be extracted. Before initiating the full data extraction process, the form was pilot-tested on a subset of studies to ensure clarity and uniform application. The parameters described in this section are organized thematically into four supplementary tables: author information (Table A1), study information (Table A2), MRI parameters (Table A3), and software details (Table A4).

## Results

In this section, the fundamental characteristics of the eligible studies are presented, offering a comprehensive overview of their demographic, anatomical, and methodological diversity. In addition, the eligible studies’ approaches for the quantification of IMF and IMAT in skeletal muscle are described in further detail. The degree of automation of these methods is evaluated, and studies are highlighted, which validate their findings with established reference standards for IMF and IMAT quantification.

### Study characteristics

**Fig. 1:**
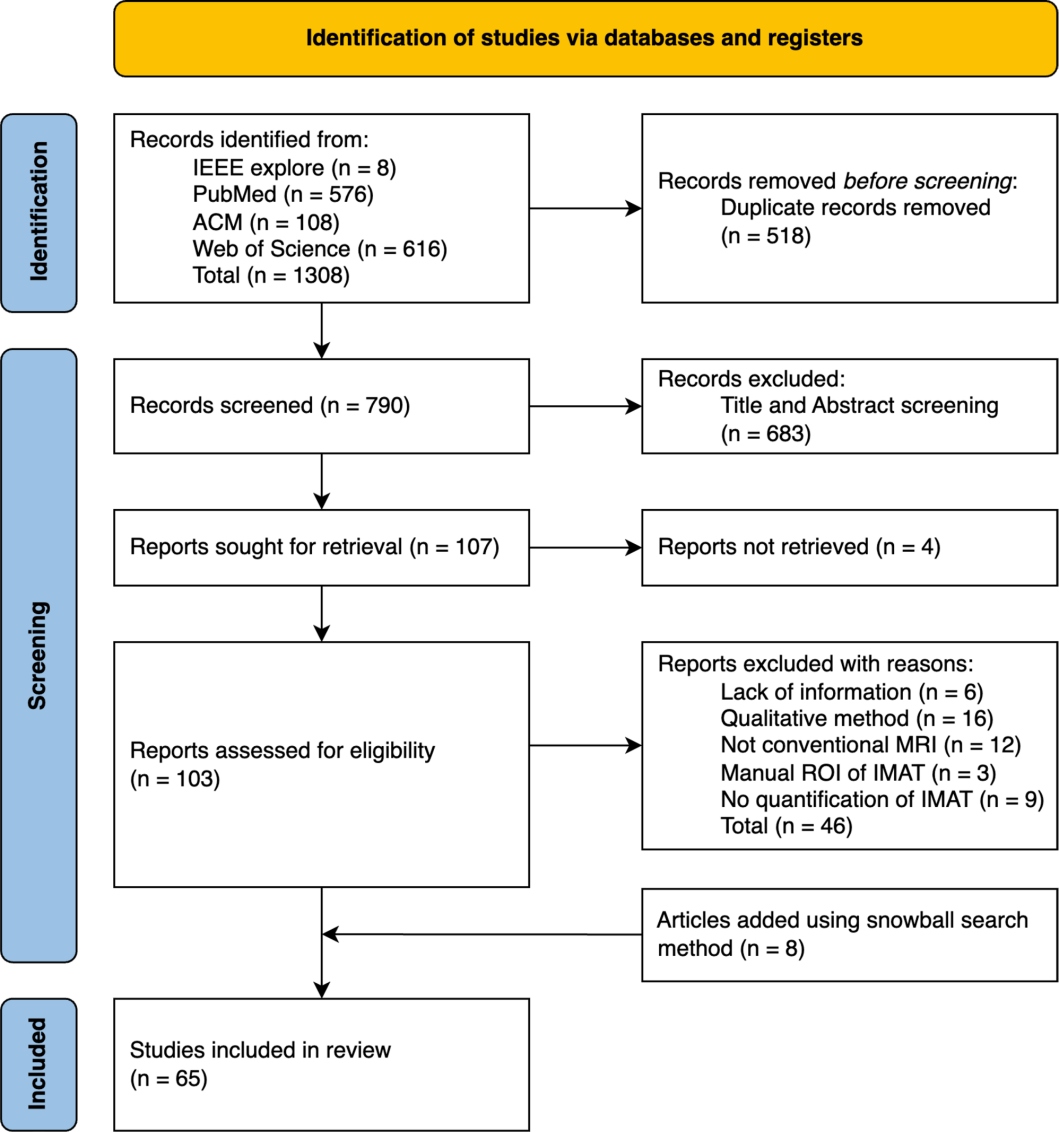
Modified prisma flowchart of the study selection process. Last update on 2024/12/05

A total of 65 studies meet the inclusion criteria after the selection process. The screening and inclusion workflow is illustrated in a PRISMA flow diagram in Fig. 1. Within the eligible studies, several noteworthy general associations were identified, contingent on the anatomical region. A number of these relationships are discussed in the following sections.

#### Study populations and anatomical focus

Of the included studies, 95% focused on humans, 3% on dogs, and 2% compared humans and sheep. Anatomical focus was primarily on the spine (68%), followed by the legs (25%), shoulder (6%), and hip (2%). Among spine studies, 89% examined the lumbar region, 7% the cervical region, and 5% the thoracic region. Leg studies predominantly investigated the thigh (63%) with 31% focusing on the calf and 6% examining both regions.

#### Muscle segmentation practices

A fundamental step in IMF and IMAT quantification on MRI scans is the segmentation of individual muscles or muscle groups, referring to the delineation of specific muscle tissue in order to isolate regions of interest for subsequent analysis. Muscle segmentation approaches varied by anatomical region. Thigh and calf studies often combined all visible muscles on MRI slices instead of segmenting individual muscles (67% of calf studies and 45% of thigh studies). In contrast, lumbar spine studies predominantly segmented individual muscles. Among the 39 lumbar spine studies, 77% segmented the multifidus, 69% the erector spinae, 51% the psoas major, and 26% employed a combined mask of the multifidus and erector spinae. Less commonly, 15% included the quadratus lumborum, and 3% examined the rectus abdominis. Detailed results are provided in (Table A2).

#### Automatic and manual segmentation techniques

Segmentation approaches span a continuum from fully manual delineation by experts to fully automated methods based on algorithmic techniques such as atlas-based registration or deep learning, differing in their degree of user involvement, reproducibility, and scalability. The segmentation techniques employed differed by anatomical region. In leg studies, 50% utilized manual segmentation, while the remainder employed automatic segmentation approaches. In spine studies, 91% relied on manual segmentation, with only 9% using automatic approaches. A detailed overview of the segmentation approaches applied across studies is available in (Table A3).

#### Imaging modalities

Imaging modalities varied among the included studies. Of the 65 studies, 46% utilized only T1-weighted imaging, 49% relied solely on T2-weighted imaging, and 5% used both modalities. T2-weighted imaging was predominantly employed in spine studies (97%), whereas T1-weighted imaging was more frequently used for leg studies (47%). All shoulder and hip studies exclusively used T1-weighted imaging, alongside 28% of spine studies.

### Quantification methods

This section provides an overview of the methods used to quantify IMF and IMAT, grouped by their methodological principles: local signal intensity-based, histogram shape-based, unsupervised, and supervised machine learning approaches.

It is worth noting that the reviewed studies varied in what they assessed: some focused on IMF, others on IMAT, and several evaluated both simultaneously. A detailed overview of which studies assessed which adipose tissue can be found in (Table A2).

In all approaches that rely on thresholding to distinguish between adipose and lean muscle tissue within a specific ROI, including both local signal intensity- and histogram shape-based methods, the proportion of either IMF or IMAT is calculated using Eq. (1). Here, a threshold value *γ* is selected from the intensity distribution of the ROI, represented by its histogram $h(\text{ROI})$. Voxels with intensity values above this threshold are classified as adipose tissue. The percentage of fat $(\text{fat}\%)$ is then computed as the relative number of voxels above the threshold in relation to the total number of voxels in the ROI:

1 $$\text{fat}\%(\text{ROI}) = \Big(\sum\limits_{i=\gamma}^{\max(\text{ROI})} h(i) \Big)/\Big(\sum\limits_{i=\min(\text{ROI})}^{\max(\text{ROI})} h(i) \Big) \cdot 100$$

The selection of *γ* can be manual, based on literature or expert knowledge, or automatic, e.g., using histogram analysis techniques.

#### Local signal intensity-based

These methodologies differ from alternative approaches in that the input signal intensities used to specify a threshold for dividing IMF or IMAT and lean muscle mass are not purely retrieved from all signal intensities of voxels inside of the delineated muscle mask. Instead, the threshold is derived from one or multiple manually selected ROIs from the same image. The selection of these ROIs is made based on the expertise of the researcher in discerning the underlying tissue characteristics, which consists of either visceral or subcutaneous fatty tissue, lean muscle tissue, bone or connective tissue. The information obtained from these designated ROIs is subsequently employed in a variety of ways on the muscle of interest. A detailed description of the 30 studies that followed this approach is provided below.

The first approach relies on manual delineating four to six RIOs within visibly identified lean muscle tissue of the muscle of interest. The highest signal intensity value observed within these RIOs is then calculated and designated as the threshold dividing lean muscle mass and the combination of IMF and IMAT in the prior segmented muscle masks of the lumbar and cervical spine. This approach can be traced back to a study by Fortin et al. [8], and has been employed in 16 studies [9–24]. A similar approach involves the manual delineation of ROIs within the visibly identified subcutaneous adipose tissue right next to the paraspinal muscles of interest [25, 26]. The lowest signal intensity within these ROIs then serves as the threshold. Some studies utilize a discrete value of 120 as a threshold [27–29]. These studies refer to the work of Ranson et al. [30], who delineated lean muscle, subcutaneous fat, and bone on multiple slices of several patients axial MRI images of the lumbar spine and calculated a threshold of 120 based on the signal intensity values of the different ROIs.

A similar approach for the lower extremities is outlined by Young and colleagues [31]. In this approach, the mean signal intensity for each visually determined tissue type is calculated by manually delineating multiple ROIs of fatty tissue, lean muscle tissue, and connective tissue. On that basis, the weighted fat percentage for each voxel in the image is calculated, which is added up to determine the IMF for a specific muscle of interest.

An alternative approach, conducted on images of the spine, entails dividing the mean signal intensity of all voxels within the boundaries of an identified muscle of interest by the mean signal intensity of a ROI comprising either subcutaneous [32–36] or visceral fatty tissue [37, 38].

Wong et al. [39] developed a method that is initially based on the gray values of a local region of the image. Subsequently, the threshold is iteratively adjusted until convergence is achieved. The framework is comprised of an initial threshold based on the fifth largest gray value of a line drawn into an MRI image of the calf, which is applied to the pre-segmented image to generate a binary mask. This mask is then alternated by removing islands of fat smaller than 16 connected voxels, a value determined empirically. A new threshold is calculated based on the average signal intensities of the identified fat and lean muscle tissue. This process is repeated until the threshold converges.

#### Histogram shape-based

The methods described in this section are all based on analyzing the shape of the histogram of the signal intensities of a ROI. It is expected that the histogram will exhibit two or three peaks in different signal intensity regions, with the algorithms attempting to find a threshold to optimally divide the expected distribution. Out of the 23 approaches belonging to this category, seven approaches can be assigned to both local signal intensity-based methods and histogram shape-based methods, as they combine both principles.

Several studies employed Otsu’s method to determine the threshold dividing lean muscle tissue and fat tissue. This algorithm identifies the threshold that divides the histogram into two classes, minimizing the intra-class intensity variance within each group. Two studies used the algorithm on the histogram of the segmented muscle of interest in MRI images of the shoulder [40, 41] and seven on the spine [42–48]. Arrieta et al. [47] used Otsu’s method, but chose to utilize Yen’s algorithm in cases where the fraction of IMF and/or IMAT exceeded 50% after using Otsu’s method. In their study, Ornowski et al. [48] implemented Otsu’s method, however, they employed it with three distinct classes. Subsequently, they determined the threshold as the arithmetic mean of the two thresholds obtained through Otsu’s method. In five studies, based on MRIs of the thigh, three ROIs of subcutaneous fat and three of lean muscle were selected [49–53]. Subsequently, Otsu’s method was applied to the histogram of the aforementioned six ROIs with the objective of determining a threshold to apply to the muscle of interest. This approach entails a combination of a histogram shape-based approach and a local signal intensity-based approach.

An alternative methodology is to fit two [48, 49, 54–58] Gaussian curves to the histogram of the muscle of interest. The point of intersection of the curves represents the threshold. This method was performed on MRIs of the spine and of the leg.

Another approach by Moser et al. [59] is the calculation of the mean and standard deviation of signal intensity values. The threshold is defined as the sum of the mean signal intensity and the standard deviation.

An additional approach by Cooley et al. [60] entails the integration of the muscle of interest’s segmentation mask with a section of subcutaneous fat surrounding the muscle of interest. This approach entails a combination of a histogram shape-based approach and a local signal intensity-based approach. In order to determine the threshold, the point at which the histogram curve intersects the x-axis is identified to the nearest value of ten in the histogram of the combined mask.

The overlap method, as proposed by Liu et al. [14], is based on the plotting of the histogram of the muscle of interest and the histogram of segmented subcutaneous back fat in a single plot. The area of intersection of both curves represents the amount of IMF and IMAT in the ROI. This approach entails a combination of a histogram shape-based approach and a local signal intensity-based approach.

#### Unsupervised machine learning

K-Means [61, 62] and Fuzzy C-Means [63] (FCM) are two clustering algorithms that have gained significant popularity in the field of image segmentation. These algorithms aim to partition data into clusters based on intensity similarity. A key feature of both K-Means and FCM is their reliance on iterative optimization processes. However, a notable distinction between these two algorithms emerges in their approach to assigning voxels to clusters. While K-Means assigns each voxel exclusively to a single cluster, FCM employs a more flexible strategy by computing membership degrees for voxels across multiple clusters, thereby accommodating overlapping intensities [64]. The key parameters that can be adjusted include the number of clusters, the initialization method of centroids, and the stopping criterion. For FCM, additional adjustments are necessary. These adjustments include the fuzziness parameter, which controls the degree of overlap between clusters, and the membership convergence threshold. These parameters influence the algorithm’s sensitivity and segmentation accuracy.

Another widely used approach is Gaussian Mixture Modelling (GMM), which assumes that the observed intensity distribution arises from a combination of underlying Gaussian components. In contrast to K-Means, which performs hard assignments, and FCM, which uses fuzzy membership values, GMM assigns voxel-specific probabilities to each Gaussian component, thus providing a probabilistic and generative framework for segmentation. Each component represents a tissue class, and the model captures the overall intensity distribution through its estimated means and covariances.

A total of eleven studies adopted clustering-based approaches with three applying K-Means, six utilizing FCM, one GMM, and one both K-Means and GMM.

K-Means was applied to images of the spine [2, 48, 65] and to images of the leg [66]. The algorithm is employed with three clusters in all studies. In the study focusing on the leg, these three clusters aim to classify bone, muscle and IMAT. In studies investigating the spine, the assignment of clusters to lean muscle and fatty tissue varies across publications. While Sasaki et al. [65] associate the two clusters with lower signal intensities with lean muscle and the highest-intensity cluster with fat, Ornowski et al. [48] define a threshold by averaging the intensity values located between the first and second, and between the second and third cluster centroids. In contrast, Wesselink et al. [2] assign the cluster with the lowest signal intensities to lean muscle, while both higher-intensity clusters are considered to represent fat. In their study, Wesselink et al. [2] not only employed K-Means with three clusters, but also explored a two-cluster variant. In addition, they applied GMM with both two and three components. For the three-class GMM, the assignment of clusters to lean muscle and fatty tissue followed the same scheme as in the three-class K-Means segmentation. This study distinguished itself by the utmost precision in its articulation of the employed centroid initialization methodology, the value of maximum iterations, and the tolerance parameter. Weber et al. [67] implemented GMM with two classes.

FCM was applied to images of the leg three times [68–70], the shoulders twice [71, 72], and the hip once [73]. Chambers et al. [68] used four clusters, with the resulting threshold being calculated as the arithmetic average of the maximum signal intensity of the second cluster and the minimum signal intensity of the fourth cluster. Orgiu et al. [70] applied the algorithm to the full image of the leg and used three clusters for bone/background, lean muscle mass, and fatty tissue/sponge bone respectively. Subsequently, this study employs active snakes evolution for the segmentation of the line separating subcutaneous fat and muscle. The amount of IMAT can then be determined based on the results of FCM in the new segmented area. The other four studies [69, 71–73] only utilized two clusters for directly distinguishing fatty tissue and lean muscle mass. All studies clearly describe the value of maximum iterations, the tolerance parameter, and the fuzziness parameter. However, the centroid initialization method remains unclear.

#### Supervised machine learning

A total of five authors utilize supervised machine learning techniques to quantify IMF or IMAT in skeletal muscle.

In their study, Yao et al. [74] employed a pre-trained (CNN), specifically VGG-16 [75], which they subsequently trained on manually segmented MRI images of the thigh. When applied to a novel image, each voxel is assigned a number between zero and one. Afterwards a threshold of 0.5 is applied to the output in order to assign each voxel as either fatty tissue or lean muscle.

Amer et al. [76] employed a patch-based deep convolutional auto-encoder to analyze images of the thigh and calf. They utilized K-Means on the learned embedding to cluster the two distinct tissue types for all muscles combined as one single ROI in the image. The use of Bloch simulations to estimate the actual echo modulation curve (EMC) and construct individual T2 and proton density maps was particularly noteworthy. Assuming that each voxel consists of a mixture of two T2 components, the signal was decomposed and the fat and water fractions within each voxel were estimated. The voxels that exhibited a (FF) greater than 50% were annotated as fat tissue, while those with lower FFs were designated as muscle tissue. This labeling scheme constituted the ground truth for the convolutional auto-encoder.

Baur et al. [77] implemented a U-Net architecture [78], a CNN predominantly used in medical image segmentation, to segment lean muscle tissue and fatty tissue from MRI images of the paraspinal muscles. The ground truth that served as the training data comprised individual threshold adaptation applied to a subset of the images.

In a study conducted by Chen et al. [79], a framework was employed that included the generation of pseudo-labels with a pre-trained neural network and the subsequent correction of these pseudo-labels. This was followed by the application of a noise-robust loss function in order to facilitate the segmentation of the lean mass of all muscles visible on MRI images of the thigh.

In a study by Ornowski et al. [48] a quadratic discriminant analysis (QDA) was conducted on T1- and T2-weighted images of the lumbar spine. The QDA represents a supervised learning approach that partitions classes through the optimization of a quadratic function. The ground truth dataset was derived from the quantified IMF amount present in six-point Dixon images and was subsequently employed to ascertain the number of voxels that required classification as fat in the T1- and T2-weighted images, respectively, in order to correspond to the IMF present in the ground truth image. The QDAs inputs comprise the signal intensity of each voxel, the mean signal intensity of a $15\times 15$ region surrounding the voxel of interest, and the mean signal intensity of the entire segmented muscle. The classification of voxels as IMF or lean muscle is the output of the QDA.

### Automation potential of methods

In this section, each approach of the eligible studies is classified according to their level of automation. Table 3 provides a comprehensive overview of the automation level, while the eligible studies are grouped by their underlying principles of the approach used. If approaches were not classified as fully automated (automation level 4) the necessary steps to achieve full automation are described.

**Table 3:** Classification of all eligible studies based on their degree of automation, organized by method-subgroups and anatomical region (anat. region) together with the number (#) of studies for each subgroup and anat. region, providing a comprehensive overview

Degree of Automation	Fat Quantification Method			
	Local-Signal Intensity-based	Histogram Shape-based	Unsupervised Machine Learning	Supervised Machine Learning
	# anat. region	# anat. region	# anat. region	# anat. region
1				
2	1 Leg [31]	5 Leg [49–53]		
	25 Spine [9–26, 32–38]	2 Spine [14, 60]		
3	3 Spine [27–29]			
4*	1 Leg [39]	3 Leg [49, 57, 58]	1 Hip [73]	3 Leg [74, 76, 79]
		2 Shoulder [40, 41]	4 Leg [66, 68–70]	1 Spine [48]
		11 Spine [42–48, 54–56, 59]	2 Shoulder [71, 72]	
			4 Spine [2, 48, 65, 67]	
4				1 Spine [77]

#### Local signal intensity-based

Among the local signal intensity-based methods, the majority, 26 studies, were assigned an automation level of 2. These methods primarily depend on user expertise to identify and segment ROIs corresponding to specific tissue types, enabling threshold determination based on the signal intensity within these ROIs. The free software ImageJ (National Institutes of Health, Bethesda, MD, USA, https://imagej.net/ij/) was the predominant software used, with other studies utilizing the commercial software OsiriX (Pixmeo, Geneva, Switzerland, https://www.osirix-viewer.com) and the commercial software Analyze (Mayo Clinic, Rochester, MN, USA, https://analyzedirect.com). Additionally, two studies implemented a MATLAB (The MathWorks, Inc., Natick, MA, USA, https://www.mathworks.com/products/matlab.html) framework (commercial software) with an integrated user interface to facilitate ROI selection. For these methods to achieve full automation, it is essential to embed the required expert knowledge into the automated selection of appropriate ROIs. In order to meet the criteria of automation level 4, batch processing capabilities need to be established to allow the analysis of multiple images at once.

Three studies were assigned an automation level of 3, all of which utilized ImageJ with a single pre-specified threshold across images to calculate the percentage of IMF or IMAT. To achieve a full automation level of 4, additional steps would be required, including automating the threshold input process and enabling batch processing capabilities for multiple images.

One study achieved an automation level of 4*, though it remains unclear whether it supports fully automated processing of multiple images.

#### Histogram shape-based

Seven studies were classified with an automation level of 2. Six out of them applied Otsu’s method to subjectively defined ROIs within specific lean muscle and subcutaneous fat areas. The requirement for expert knowledge to select ROIs in precise regions of the image limits the automation level, as manual intervention is necessary. To achieve a level 4* automation, these methods would need to incorporate fully automated processes for identifying distinct ROIs within the designated areas of the image. Further advancement to level 4 would require the implementation of a batch-processing routine capable of handling multiple images sequentially. The majority of these studies utilized the free software MIPAV (National Institutes of Health, Bethesda, MD, USA, https://mipav.cit.nih.gov) and commercial software Amira (Thermo Fisher Scientific Inc., Waltham, USA, https://www.thermofisher.com/de/de/home/electron-microscopy/products/software-em-3d-vis/amira-software.html), both of which support medical imaging tasks but currently lack integrated solutions for fully automated processing in this context. The final study, classified with an automation level of 2, utilized the commercial software SliceOmatic (TomoVision, Magog, Canada, https://www.tomovision.com/products/sliceomatic.html) to identify the zero-crossing point of a smoothed histogram for the ROI, which included both multifidus muscles and a connecting bridge of subcutaneous fat. In this method, the chosen threshold serves as the primary output, but several steps are necessary to achieve full automation. Specifically, the automated determination of the ROI would require precise specifications regarding the amount of fatty tissue to include, as this selection critically influences the threshold calculation. Additionally, the zero-crossing point should be detected automatically to ensure consistency. For a complete transition to level 4 automation, batch processing capabilities must be established to allow the concurrent analysis of multiple images.

The remaining 17 studies were assigned an automation level of 4*, employing Otsu’s method, Yen’s method, or Gaussian fitting techniques. Most of these studies implemented their algorithms in MATLAB or Python (free software), with additional software including the free software MeVisLab (MeVis Medical Solutions AG and Fraunhofer MEVIS, Bremen, Germany, http://www.mevislab.de/), MIPAV, and OsiriX. According to the study descriptions, these methods generally operate automatically. However, to achieve a full automation level of 4, batch-processing capabilities for handling multiple images concurrently would needed to be established.

#### Unsupervised machine learning

All ten studies utilizing unsupervised machine learning approaches were assigned an automation level of 4*, reflecting substantial automation, but with uncertain capacity for batch processing. The methods were primarily developed using either MATLAB, Python, or MIPAV.

#### Supervised machine learning

This section examines the degree of automation and the requisite steps for achieving full automation for all methods employing supervised machine learning approaches for the quantification of IMF or IMAT. The majority of these studies were implemented in Python. However, one study did not specify the programming language used. Four of the five studies achieved an automation level of 4*, with one study reaching full automation. The implementation of a pipeline that allows for sequential image processing and result storage would be necessary to achieve level 4 automation.

### Validity of methods

The following section discusses studies that have made comparisons between their results and a reference standard, as defined in the methods section. In total, nine out of the eligible 65 studies fulfilled this criterion. The remaining studies either made comparisons between their results and a semi-quantitative score or different quantification methods not based on MRI, did not report any comparison, or focused on the reliability of their results by determining the intra- and interobserver agreement.

Among the nine validation studies, MRS, Two-point Dixon, and Six-point Dixon MRI were used as a reference standard. While MRS is widely regarded as the most accurate technique for quantifying FF, its limited spatial coverage necessitates careful alignment between the MRS voxel and MRI-based ROIs to avoid misleading comparisons. Six-point Dixon MRI, by contrast, provides voxel-wise FF estimates and has demonstrated high correlation with MRS [80]. Two-point Dixon sequences are comparable only if dedicated echo timings are applied [80].

The identified validation studies show considerable heterogeneity in their methodological design, selected regions of interest and anatomical focus, as well as in the qMRI reference standards used. In addition, a wide variety of statistical approaches were applied to assess agreement. In the following, the studies’ findings and conclusions are summarized qualitatively. Explicit numerical results are reported only for the intraclass correlation coefficient (ICC) and Pearson’s correlation coefficient, as these were consistently available across all nine studies. A direct quantitative comparison of outcomes is not performed, since the nine studies differ considerably in their design, methodology, and contextual parameters, precluding exact comparability.

#### Validation studies of the paraspinal muscles

Several studies have investigated the validity and methodological performance of approaches for quantifying IMF or the combination of IMF and IMAT in paraspinal muscles using conventional MRI sequences.

Table 4 presents an overview of these studies. The first column provides the citation reference (Ref.), followed by columns for the number of subjects (N), the analyzed spinal levels (top) and the investigated muscles (bottom), the MRI method showing the sequence type (T1- or T2-weighted) and applied segmentation or classification approach, the quantitative MRI reference method, and the degree of automation. Information about the level of correlation or agreement between automated and reference measurements as stated in the respective studies is shown in the bottom part in the column Agreement (Category). For measuring the agreement, the respective studies use the Pearson correlation coefficient (r) or the ICC with qualitative descriptors (e.g., Excellent, Strong, Moderate, Poor). We abbreviate the spinal muscles as multi-fidus (MF), erector spinae (ES), psoas major (PM) and quadratus lumborum (QL). The segmentation or classification methods use the abbreviations 2C/3C = two/three classes.

**Table 4:** Summary of studies investigating (semi-)automated MRI-based approaches for quantifying lumbar paraspinal muscle composition, validated against established reference standards. A detailed explanation of the column headers are explained in the section discussing the validated lumbar paraspinal studies (see Section 3)

Ref.	N	Spinal Levels	MRI Method	Quantitative MRI Ref.	Autom. Degree
		Muscles	Agreement (Category)		
[47]	37	L1–S1	T1; Otsu or Yen	2-point Dixon	4*
		MF, ES	MF+ES: r 0.96 (Excellent)		
[11]	35	L4-5, L5–S1	T2; 4–6 ROIs	2-point Dixon	2
		MF, ES, PM	MF: r 0.89–0.92 (Strong); ES: r 0.87–0.92 (Strong); PM: r −0.027–0.67 (No-Moderate)		
[48]	11	L1–S1	T1; Gaussian curve fitting	6-point Dixon	4*
		MF, ES, PM, QL	MF: r 0.72 (Strong); ES: r 0.72 (Strong); PM: r 0.31 (Weak-Moderate); QL: r 0.43 (Weak-Moderate)		
[48]	11	L1–S1	T1; Otsu	6-point Dixon	4*
		MF, ES, PM, QL	MF: r 0.71 (Strong);ES: r 0.84 (Strong); PM: r 0.23 (Weak-Moderate); QL: r 0.36 (Weak-Moderate)		
[48]	11	L1–S1	T1; QDA	6-point Dixon	4*
		MF, ES, PM, QL	MF: r 0.78 (Strong); ES: r 0.80 (Strong); PM: r 0.31 (Weak-Moderate); QL: r 0.44 (Weak-Moderate)		
[48]	11	L1–S1	T1; K-means 3C	6-point Dixon	4*
		MF, ES, PM, QL	MF: r 0.66 (Strong); ES: r 0.83 (Strong); PM: r 0.16 (Weak-Moderate); QL: r 0.29 (Weak-Moderate)		
[48]	11	L1–S1	T2; Gaussian curve fitting	6-point Dixon	4*
		MF, ES, PM, QL	MF: r 0.80 (Strong); ES: r 0.84 (Strong); PM: r 0.44 (Weak-Moderate); QL: r 0.35 (Weak-Moderate)		
[48]	11	L1–S1	T2; Otsu	6-point Dixon	4*
		MF, ES, PM, QL	MF: r 0.79 (Strong); ES: r 0.89 (Strong); PM: r 0.39 (Weak-Moderate); QL: r 0.48 (Weak-Moderate)		
[48]	11	L1–S1	T2; QDA	6-point Dixon	4*
		MF, ES, PM, QL	MF: r 0.72 (Strong); ES: r 0.80 (Strong); PM: r 0.29 (Weak-Moderate); QL: r 0.44 (Weak-Moderate)		
[48]	11	L1–S1	T2; K-means 3C	6-point Dixon	4*
		MF, ES, PM, QL	MF: r 0.73 (Strong); ES: r 0.87 (Strong); PM: r 0.30 (Weak-Moderate); QL: r 0.32 (Weak-Moderate)		
[2]	30	L4–S1	T2; GMM 2C	2-point Dixon	4*
		MF, ES, PM	MF: ICC 0.68–0.74 (Moderate); ES: ICC 0.63–0.70 (Moderate); PM: ICC −0.09–0.01 (Poor)		
[2]	30	L4–S1	T2; K-means 2C	2-point Dixon	4*
		MF, ES, PM	MF: ICC 0.32–0.37 (Poor); ES: ICC 0.37–0.38 (Poor); PM: ICC −0.14–0.10 (Poor)		
[2]	30	L4–S1	T2; GMM 3C	2-point Dixon	4*
		MF, ES, PM	MF: ICC 0.77–0.83 (Good); ES: ICC 0.81–0.88 (Good); PM: ICC −0.01–0.01 (Poor)		
[2]	30	L4–S1	T2; K-means 3C	2-point Dixon	4*
		MF, ES, PM	MF: ICC 0.68–0.74 (Moderate-Good); ES: ICC 0.78–0.82 (Moderate-Good); PM: ICC −0.01 (Poor)		

Arrieta et al. [47] demonstrated that FF values derived from automatically thresholded T2-weighted images showed excellent agreement with Dixon-based reference measurements (*r* = 0.86), indicating that T2-weighted imaging can provide equivalent quantitative information when appropriate thresholding algorithms are applied. Their hybrid approach, combining Otsu’s and Yen’s method, allegedly compensated for the limitations of each algorithm at different fat infiltration levels. Masi et al. [11] further confirmed the strong linear relationship between fat quantification obtained from T2-weighted and two-point Dixon MRI, particularly for the MF and ES muscles. However, they reported considerable side-to-side variability, which may reflect differences in local magnetic susceptibility and signal ambiguity, highlighting the need for methodological consistency in image acquisition and analysis. Building on these findings, Ornowski et al. [48] compared several automated thresholding methods across paraspinal muscles and MRI sequences, reporting correlation coefficients above 0.70 for all muscles, with the highest accuracy for the T2-Otsu method (*r* = 0.86). Their results suggest that method performance depends strongly on both muscle composition and the underlying signal variance, with muscles containing greater fat fractions (e.g., ES, MF) yielding more reliable estimations than those with lower fat content (e.g., PM, QL). Most recently, Wesselink et al. [2] evaluated multiple automated models and found that a three-component GMM provided the best balance of accuracy and reliability compared to other thresholding approaches. Contrary to Ornowski et al. [48], their study emphasized that algorithmic configuration, such as the number of components and initialization procedures, substantially affects IMF quantification outcomes. They also identified scanner-related factors (e.g., vendor, bit depth, intensity correction) and differences in field-of-view as potential contributors to methodological discrepancies across studies. Collectively, these investigations underscore that both imaging parameters and algorithmic design critically determine the validity and reproducibility of MRI-based IMF assessments, and they highlight the necessity for standardized acquisition and analysis protocols in future research.

#### Validation studies of the thigh

The validity and methodological performance of approaches for quantifying IMF in muscles of the thigh using conventional MRI sequences has been investigated in three studies, which are summarized in Table 5. Apart from the study citation reference (Ref.), the table shows the number of subjects (N), the investigated muscles, the MRI acquisition and segmentation method, and the used quantitative MRI reference standard (qMRI Ref.). The last column shows the degree of automation and the second row of each entry shows the reported agreement between proposed methods and the reference methods. Agreement values (Pearson correlation coefficient (r) and ICC) are provided with their qualitative descriptors reproduced as reported in the original publications. The thigh muscles analyzed are vastus lateralis (VL), vastus medialis (VM), vastus intermedius (VI), rectus femoris (RF), adductor magnus (AM), biceps femoris (BF), biceps femoris long head (BF-L), biceps femoris short head (BF-S), semitendinosus (ST) and semimembranosus (SM).

**Table 5:** Summary of studies investigating (semi-)automated MRI-based approaches for quantifying thigh muscle composition, validated against established reference standards. A detailed explanation of the column headers are explained in the section discussing the validated thigh studies (see Section 3)

Ref.	N	Muscles	MRI Method	qMRI Ref.	Autom. Degree
			Agreement (Category)		
[52]	30	VL, BF	T1; Otsu on 6 25 mm*25 mm ROIs	MRS	2
			EMCL VL r 0.506 (Significant corr.), IMCL VL r 0.263 (No corr.), EMCL BF r 0.590 (Significant corr.), IMCL BF r 0.236 (No corr.)		
[50]	32	VL, AM, BF-L	T1; Otsu on 6 25 mm*25 mm ROIs	2-point Dixon	2
			VL r 0.735 (High), AM r −0.717 (High), BF-L r 0.790 (High), VL+AM+BF-L r 0.686 (High)		
[69]	9	VL, VM, VI, RF, BF-L, BF-S, ST, SM	T1; FCM	2-point Dixon	4*
			ICC 0.81–0.93 (Good)		
[69]	11	VL, VM, VI, RF	T1; FCM	2-point Dixon	4*
			N/A		

Akima et al. [52], using MRS as reference standard, demonstrated that MRI-derived IMF content was significantly correlated with EMCL but not with IMCL, indicating that methods based on conventional MRI primarily reflect lipid accumulation outside, rather than inside muscle fibers. Ogawa et al. [50] compared T1-weighted and two-point Dixon imaging for estimating IMF in the AM, BF-L, and VL. They found that IMF content was significantly higher when measured with T1-weighted MRI than with two-point Dixon imaging for the AM and BF-L, whereas no difference was observed for the VL. These discrepancies were attributed to systematic thresholding differences between methods, highlighting that muscle-specific contrast and threshold choice can substantially affect quantitative outcomes. Similarly, Bolsterlee et al. [69] evaluated T1-weighted MRI and two-point Dixon imaging across a wide range of fat proportions (2–76%) and found that T1-weighted MRI was unreliable in relatively lean muscles (below ~8% fat), as neither manual nor fuzzy C-means classification could identify a distinct boundary between muscle and fat. However, in muscles with higher fat content, both methods yielded comparable results (ICC = 0.88) with no systematic bias, though T1-weighted imaging tended to slightly underestimate fat at very high infiltration levels. Collectively, these findings indicate that the accuracy and applicability of MRI-based fat quantification in thigh muscles depend strongly on muscle-specific fat composition, imaging sequence, and thresholding technique. The applied T1-weighted approaches show limited robustness, particularly in lean muscle tissue.

#### Validation studies of the shoulder and upper back muscles

Two studies have investigated the validity and methodological performance of approaches for quantifying IMF or the combination of IMF and IMAT in shoulder and upper back muscles using conventional MRI sequences. Table 6 summarizes aspects of these studies. Columns indicate the study citation reference (Ref.), number of subjects (N), investigated muscles (supraspinatus (SS), infraspinatus (IS), subscapularis (SC), teres minor (TM), deltoid (D), trapezius (T) and supraspinatus fossa (SF)), MRI acquisition and segmentation method, quantitative MRI reference method (qMRI Ref.), and degree of automation. The agreement between proposed methods and reference measurements is shown in the bottom part of each entry (using Pearson correlation coefficient (r) or coefficient of determination (*R*^2^)) with qualitative categories. The agreement values and qualitative descriptors are reproduced exactly as reported in the original studies.

**Table 6:** Summary of studies investigating automated MRI-based approaches for quantifying shoulder and upper back muscle composition, validated against established reference standards. A detailed explanation of the column headers are explained in the section discussing the validated shoulder studies (see Section 3)

Ref.	N	Muscles	MRI Method	qMRI Ref.	Autom. Degree
			Agreement (Category)		
[72]	7	SS, IS, SC, TM, D, T	T1; FCM	6-point Dixon	4*
			All: r 0.85 (Strong)		
[40]	38	SS, SF	T1; Otsu	MRS	4*
			SS: *R*^2^ 0.83 (Strong); SF: *R*^2^ 0.68 (Moderate)		

Davis et al. [72] demonstrated that fuzzy C-means segmentation performed on standard T1-weighted images correlated strongly with fat fraction measurements derived from six-point Dixon MRI. Similarly, Lee et al. [40] applied a threshold-based segmentation approach to quantify fat fractions in the SS muscle and SF. Their results showed strong correlations between threshold-based fat fractions and the fat/water ratios obtained from MRS (R^2^ = 0.83) for the SS, suggesting that T1-weighted MRI can capture intramuscular fat variations with reasonable accuracy. However, correlations were only moderate for the SF, likely due to the limited spatial sampling of MRS, which measures fat content within a single voxel. Overall, these studies indicate that both fuzzy C-means and threshold-based methods can provide valid estimates of shoulder muscle fat infiltration on conventional MRI. Nonetheless, variations in segmentation strategy, anatomical focus, and reference techniques preclude direct quantitative comparison of results across studies.

## Discussion

The presented review follows the PRISMA guidelines, uses a PICO-based guiding question, and uses a systematically constructed search string as well as in- and exclusion criteria based upon the guiding question. This ensures a structured, transparent framework for identifying relevant studies. However, these stringent criteria may exclude studies that do not align perfectly with the defined parameters, potentially leading to the omission of valuable insights. Despite this limitation, the use of such rigorous guidelines is highly recommended, as it ensures reproducibility, clarity, and methodological consistency in literature reviews, contributing to the reliability and integrity of the research process.

This study is subject to certain limitations due to the scope and focus of the reviewed approaches. For instance, the study does neither include semi-quantitative classification for evaluating IMF or IMAT nor does it encompass other imaging modalities such as ultrasound, CT, and quantitative MRI alone, or assessments of fat quantification in tissues beyond skeletal muscle such as hepatic fat in the liver. These exclusions are deliberate in order to maintain a focus on conventional MRI-based approaches in skeletal muscle research. Another limitation arises from the substantial heterogeneity in statistical analyses between studies and incomplete reporting of results, both of which pose challenges to direct comparisons and limit the generalizability of findings.

Despite these constraints, the study offers a comprehensive overview by grouping methodologies according to underlying concepts, providing information on the dominant methodological trends and innovations in the field over the past decade. It also differentiates methods based on the anatomical regions and assesses and systematically evaluates the degree of automation that each approach achieves. Importantly, the study highlights publications that validate their results against reference standards, underscoring the value of methodological rigor and reliability. In doing so, this review not only offers an organized synthesis of recent advancements, but also identifies pathways towards more fully automated and standardized methodologies in IMF and IMAT assessment.

## Conclusions and future directions

In conclusion, the analysis of IMF and IMAT from conventional MRI requires careful methodological selection. We recommend that initial methodological choices be guided by the current literature, prioritizing studies that analyze similar muscle groups, anatomical regions, and MRI parameters. Moreover, we recommend employing methodologies derived from studies using a reference standard for the comparison of their results. Such comparisons allow users to estimate methodological accuracy and understand potential error margins. While current evidence does not provide comprehensive comparative performance data across automation levels, methods assessed with a high degree of automation (levels 4 or 4*) may still be considered advantageous from a practical standpoint. Their benefits, such as improved reproducibility, scalability, and integration potential, are particularly relevant for routine clinical use and for the analysis of large datasets, where manual intervention is neither feasible nor desirable. However, users should remain aware that these practical advantages do not necessarily imply superior quantitative accuracy, as direct performance comparisons are still lacking. As this review does not permit performance-based recommendations regarding IMF or IMAT quantification results, we encourage users to consult validated literature for guidance or to compare a representative subset of their own data against one of the established reference standards to ensure methodological reliability.

For future research, particularly in prospective studies, the establishment of standardized MRI protocols and transparent, consistent guidelines for both analysis and result reporting are essential. Such standards would facilitate comparisons across research groups, enhancing the reproducibility and reliability of findings. Additionally, extensive studies are warranted to investigate the impact of diverse methodologies, MRI parameters, and preprocessing approaches on outcomes, and to benchmark these results against a broader range of reference standard methods. These investigations would deepen our understanding of the diverse sources of error affecting the quantification of IMF and IMAT in conventional MRI images, paving the way for more accurate and comparable analyses across studies. By fostering the adoption of validated, automated approaches, future research can ensure that IMF and IMAT quantification using conventional MRI becomes a reliable, accessible, and clinically relevant tool. This transition has the potential to improve early diagnosis, enhance disease monitoring, and contribute to more informed clinical decision-making in musculoskeletal health.

## Data Availability

All data generated or analyzed during this study are included in this published article.

## Appendix A: Structured information about the eligible studies

This appendix provides structured supplementary information on all studies included in this review. Table A1 summarizes bibliographic details, including reference identifiers, DOIs, and author affiliations. Table A2 presents study-level characteristics such as the examined body region, segmented muscles, adipose tissue assessed, species, and cohort size. Table A3 outlines MRI acquisition and analysis parameters, including preprocessing, segmentation, and fat quantification methods. Table A4 details software-related aspects, covering software and code availability, quantification categories, automation degree, and validation standards.

**Table A1:** Overview of bibliographic information for the surveyed studies. The table summarizes key publication details of the included studies. “DOI” provides the corresponding digital object identifier. “Last Author” and “Country Affiliation Last Author” indicate the senior author and their institutional country, respectively

Ref.	Title			
	DOI			
	First Author	Last Author	Country Affiliation Last Author	Year Published
[52]	*Intramuscular Adipose Tissue Determined by T1-Weighted MRI at 3T Primarily Reflects Extramyocellular Lipids.*			
	10.1016/j.mri.2015.12.038			
	Akima, Hiroshi	Oshida, Yoshiharu	Japan	2016
[53]	*Skeletal muscle size is a major predictor of intramuscular fat content regardless of age.*			
	10.1007/s00421-015–3148-2			
	Akima, Hiroshi	Oshida, Yoshiharu	Japan	2015
[76]	*Automatic Segmentation of Muscle Tissue and Inter-Muscular Fat in Thigh and Calf MRI Images.*			
	10.1007/978–3-030–32245-8_25			
	Amer, Rula	Ben-Eliezer, Noam	USA, Israel	2019
[47]	*Automatic Quantification of Fat Infiltration in Paraspinal Muscles Using T2-Weighted Images: An OsiriX Application.*			
	10.1016/J.BSPC.2019.101793			
	Arrieta, Cristobal	Uribe, Sergio	Chile	2020
[77]	*Analysis of the Paraspinal Muscle Morphology of the Lumbar Spine Using a Convolutional Neural Network (CNN).*			
	10.1007/s00586-021–07073-y			
	Baur, David	Voelker, Anna	Germany	2022
[55]	*Methodological Considerations in Region of Interest Definitions for Paraspinal Muscles in Axial MRIs of the Lumbar Spine.*			
	10.1186/s12891-018–2059-x			
	Berry, David B.	Shahidi, Bahar	USA	2018
[69]	*MRI-Based Measurement of Effects of Strength Training on Intramuscular Fat in People with and without Spinal Cord Injury.*			
	10.1249/MSS.0000000000002568			
	Bolsterlee, Bart	Herbert, Robert D.	Australia	2021
[34]	*Comparison of cross sectional area and fat infiltration of the epaxial muscles in dogs with and without spinal cord compression.*			
	10.1016/j.rvsc.2014.09.006			
	Boström, Anna F.	Davies, Emma S.	UK	2014
[35]	*Epaxial muscle atrophy is more evident in large dogs with intervertebral disc disease than in dogs with ischaemic myelopathy.*			
	10.1016/j.rvsc.2022.03.011			
	Boström, Anna F.	Davies, Emma S.	UK	2022
[68]	*Computer-Based Assessment for Facioscapulohumeral Dystrophy Diagnosis.*			
	10.1016/j.cmpb.2015.03.006			
	Chambers, O.	Tasči, J. F.	Slovenia	2015
[79]	*Precise Few-Shot Fat-Free Thigh Muscle Segmentation in T1-Weighted MRI.*			
	10.48550/ARXIV.2304.14053			
	Chen, Sheng	Cai, Weidong	Australia	2023
[60]	*Assessing Lumbar Paraspinal Muscle Cross-Sectional Area and Fat Composition with T1 versus T2-Weighted Magnetic Resonance Imaging: Reliability and Concurrent Validity.*			
	10.1371/journal.pone.0244633			
	Cooley, J. R.	Walker, B. F.	Australia	2021
[26]	*Geography of Lumbar Paravertebral Muscle Fatty Infiltration: The Influence of Demographics, Low Back Pain, and Disability.*			
	10.1097/BRS.0000000000003060			
	Crawford, Rebecca J.	Samartzis, Dino	USA	2019
[72]	*Quantification of Shoulder Muscle Intramuscular Fatty Infiltration on T1-Weighted MRI: A Viable Alternative to the Goutallier Classification System.*			
	10.1007/s00256-018–3057-7			
	Davis, Derik L.	Zhuo, Jiachen	USA	2019
[73]	*Gluteal Muscle Fatty Infiltration, Fall Risk, and Mobility Limitation in Older Women with Urinary Incontinence: A Pilot Study.*			
	10.1007/s00256-022–04132-3			
	Davis, Derik L.	Sanses, Tatiana V. D.	USA	2023
[71]	*Reliability of Supraspinatus Intramuscular Fatty Infiltration Estimates on T1-Weighted MRI in Potential Candidates for Rotator Cuff Repair Surgery: Full-Thickness Tear versus High-Grade Partial-Thickness Tear.*			
	10.1007/s00256-021–03805-9			
	Davis, Derik L.	Hasan, S. Ashfaq	USA	2021
[58]	*A Non-Invasive Method for a Quantitative Evaluation of Muscle Involvement in MRI of Neuromuscular Diseases Retrospective Study and Future Perspectives.*			
	Fantacci, Maria Evelina	Tosetti, Michela	Italy	2015
[57]	*Quantitative Scoring of Muscle Involvement in MRI of Neuromuscular Diseases.*			
	10.5220/0005255801000105			
	Fantacci, Maria Evelina	Tosetti, Michela	Italy	2015
[16]	*Do Variations in Paraspinal Muscle Morphology and Composition Predict Low Back Pain in Men?*			
	10.1111/sms.12301			
	Fortin, Maryse	Battié, Michele C.	Canada	2015
[22]	*Relationship between Cervical Muscle Morphology Evaluated by MRI, Cervical Muscle Strength and Functional Outcomes in Patients with Degenerative Cervical Myelopathy.*			
	10.1016/j.msksp.2018.07.003			
	Fortin, Maryse	Weber, Michael H.	Canada	2018
[15]	*Paraspinal Muscle Morphology and Composition: A 15-Yr Longitudinal Magnetic Resonance Imaging Study.*			
	10.1249/MSS.0000000000000179			
	Fortin, Maryse	Battié, Michele C.	Canada	2014
[17]	*Paraspinal Muscle Asymmetry and Fat Infiltration in Patients with Symptomatic Disc Herniation.*			
	10.1007/s00586-016–4503-7			
	Fortin, Maryse	Battié, Michele C.	Canada	2016
[13]	*Association Between Paraspinal Muscle Morphology, Clinical Symptoms, and Functional Status in Patients With Degenerative Cervical Myelopathy.*			
	10.1097/BRS.0000000000001704			
	Fortin, Maryse	Weber, Michael H.	Canada	2017
[43]	*Evaluation of an Automated Thresholding Algorithm for the Quantification of Paraspinal Muscle Composition from MRI Images.*			
	10.1186/s12938-017–0350-y			
	Fortin, Maryse	Rivaz, Hassan	Canada	2017
[12]	*Association between Paraspinal Muscle Morphology, Clinical Symptoms and Functional Status in Patients with Lumbar Spinal Stenosis.*			
	10.1007/s00586-017–5228-y			
	Fortin, Maryse	Battié, Michele C.	Canada	2017
[10]	*Quantitative Magnetic Resonance Imaging Analysis of the Cervical Spine Extensor Muscles: Intrarater and Interrater Reliability of a Novice and an Experienced Rater.*			
	10.4184/asj.2018.12.1.94			
	Fortin, Maryse	Weber, Michael H.	Canada	2018
[54]	*Age-Related Structural and Functional Changes of Low Back Muscles.*			
	10.1016/j.exger.2015.02.016			
	Hiepe, Patrick	Reichenbach, Jürgen R.	Germany	2015
[9]	*The Association between Paraspinal Muscle Degeneration and Osteoporotic Vertebral Compression Fracture Severity in Postmenopausal Women.*			
	10.3233/BMR-220059			
	Huang, Wei	Tu, Min	China	2023
[24]	*Paraspinal Muscle Fatty Degeneration as a Predictor of Progressive Vertebral Collapse in Osteoporotic Vertebral Compression Fractures.*			
	10.1016/j.spinee.2021.07.020			
	Jeon, Ikchan	Yu, Dongwoo	South Korea	2022
[29]	*Potential Role of Paraspinal Musculature in the Maintenance of Spinopelvic Alignment in Patients With Adult Spinal Deformities.*			
	10.1097/BSD.0000000000000862			
	Katsu, Marina	Haro, Hirotaka	Japan	2020
[20]	*Significance of Paraspinal Muscle Quality in Risk between Single and Multiple Osteoporotic Vertebral Fractures.*			
	10.1007/s00586-023–07670-z			
	Kim, Hong Jin	Kim, Young-Hoon	South Korea	2023
[66]	*Muscle Quantitative MR Imaging and Clustering Analysis in Patients with Facioscapulohumeral Muscular Dystrophy Type 1.*			
	10.1371/journal.pone.0132717			
	Lareau-Trudel, Emilie	Salort-Campana, Emmanuelle	France	2015
[56]	*Morphology versus Function: The Relationship between Lumbar Multifidus Intramuscular Adipose Tissue and Muscle Function among Patients with Low Back Pain.*			
	10.1016/j.apmr.2014.04.019			
	Le Cara, Edward C.	Hebert, Jeffrey J.	Australia	2014
[40]	*Threshold-Based Quantification of Fatty Degeneration in the Supraspinatus Muscle on MRI as an Alternative Method to Goutallier Classification and Single-Voxel MR Spectroscopy.*			
	10.1186/s12891-020–03400-4			
	Lee, Dokwan	Song, Yongnam	South Korea	2020
[21]	*Association of Lumbar Paraspinal Muscle Morphometry with Degenerative Spondylolisthesis.*			
	10.3390/ijerph18084037			
	Lee, Eun Taek	Chon, Jinmann	South Korea	2021
[27]	*Association between Paraspinal Muscle Fat Infiltration and Regional Kyphosis Angle in Thoracolumbar Fracture Patients: A Retrospective Study.*			
	10.1038/s41598-024–53017-z			
	Liao, Yitao	Zhang, Xian	China	2024
[14]	*Inter-Software and Inter-Threshold Reliability of Quantitative Paraspinal Muscle Segmentation.*			
	10.1007/s00586-023–08050-3			
	Liu, Sihai	Becker, Luis	Germany	2024
[18]	*Quantification of Intramuscular Fat in Patients with Late-Onset Pompe Disease by Conventional Magnetic Resonance Imaging for the Long-Term Follow-up of Enzyme Replacement Therapy.*			
	10.1371/journal.pone.0190784			
	Lollert, André	Staatz, Gundula	Germany	2018
[19]	*Assessing Fatty Infiltration of Paraspinal Muscles in Patients With Lumbar Spinal Stenosis: Goutallier Classification and Quantitative MRI Measurements.*			
	10.3389/fneur.2021.656487			
	Mandelli, Filippo	Netzer, Cordula	Switzerland	2021
[11]	*Comparison of Paraspinal Muscle Composition Measurements Using IDEAL Fat-Water and T2-Weighted MR Images.*			
	10.1186/S12880-023–00992-W			
	Masi, Sara	Fortin, Maryse	Canada	2023
[25]	*Reliability of Quantifying the Spatial Distribution of Fatty Infiltration in Lumbar Paravertebral Muscles Using a New Segmentation Method for T1-Weighted MRI.*			
	10.1186/s12891-016–1090-z			
	Mhuiris, Áine Ni	Crawford, Rebecca J.	Australia, Switzerland	2016
[28]	*The Fatty Degeneration of the Lumbar Erector Spinae Muscles Affects Dynamic Spinal Compensation Ability during Gait in Adult Spinal Deformity.*			
	10.1038/s41598-021–97358-5			
	Miura, Kousei	Yamazaki, Masashi	Japan	2021
[59]	*The predictive value of psoas and paraspinal muscle parameters measured on MRI for severe cage subsidence after standalone lateral lumbar interbody fusion*			
	10.1016/j.spinee.2022.03.009			
	Moser, Manuel	Hughes, Alexander P.	USA	2023
[44]	*An Externally Validated Deep Learning Model for the Accurate Segmentation of the Lumbar Paravertebral Muscles.*			
	10.1007/s00586-022–07320-w			
	Niemeyer, Frank	Wilke, Hans-Joachim	Germany	2022
[49]	*Quantification of Intermuscular and Intramuscular Adipose Tissue Using Magnetic Resonance Imaging after Neurodegenerative Disorders.*			
	10.4103/1673–5374.221170			
	Ogawa, Madoka	Gorgey, Ashraf S.	USA	2017
[50]	*Comparing Intramuscular Adipose Tissue on T1-Weighted and Two-Point Dixon Images.*			
	10.1371/journal.pone.0231156			
	Ogawa, Madoka	Akima, Hiroshi	Japan	2020
[33]	*Long-Term Impacts of Brace Treatment for Adolescent Idiopathic Scoliosis on Body Composition, Paraspinal Muscle Morphology, and Bone Mineral Density.*			
	10.1097/BRS.0000000000003069			
	Ohashi, Masayuki	Endo, Naoto	Japan	2019
[70]	*Automatic Muscle and Fat Segmentation in the Thigh from T1-Weighted MRI.*			
	10.1002/jmri.25031			
	Orgiu, Sara	Rizzo, Giovanna	Italy	2016
[48]	*Thresholding Approaches for Estimating Paraspinal Muscle Fat Infiltration Using T1- and T2-Weighted MRI: Comparative Analysis Using Water-Fat MRI.*			
	10.1002/jsp2.1301			
	Ornowski, Jessica	Bailey, Jeannie F.	USA	2024
[23]	*Asymmetric Atrophy of the Multifidus in Persons with Hemiplegic Presentation Post-Stroke.*			
	10.1080/10749357.2020.1846932			
	Park, Wookyung	Min, Kyunghoon	South Korea	2021
[65]	*MRI-Defined Paraspinal Muscle Morphology in Japanese Population: The Wakayama Spine Study.*			
	10.1371/journal.pone.0187765			
	Sasaki, Takahide	Yoshida, Munehito	Japan	2017
[45]	*A Deep-Learning-Based, Fully Automated Program to Segment and Quantify Major Spinal Components on Axial Lumbar Spine Magnetic Resonance Images.*			
	10.1093/ptj/pzab041			
	Shen, Haotian	Wang, Yue	China	2021
[38]	*Trunk Muscle Size and Composition Assessment in Older Adults with Chronic Low Back Pain: An Intra-Examiner and Inter-Examiner Reliability Study.*			
	10.1093/pm/pnv023			
	Sions, Jaclyn Megan	Elliott, James Matthew	USA	2016
[42]	*Lumbar Paraspinal Muscle Fat Infiltration Is Independently Associated with Sex, Age, and Inter-Vertebral Disc Degeneration in Symptomatic Patients.*			
	10.1007/s00256-018–2880-1			
	Urrutia, Julio	Uribe, Sergio	Chile	2018
[46]	*Is a Single-Level Measurement of Paraspinal Muscle Fat Infiltration and Cross-Sectional Area Representative of the Entire Lumbar Spine?*			
	10.1007/s00256-018–2902-z			
	Urrutia, Julio	Uribe, Sergio	Chile	2018
[36]	*MRI-Determined Lumbar Muscle Morphometry in Man and Sheep: Potential Biomechanical Implications for Ovine Model to Human Spine Translation.*			
	10.1111/joa.12354			
	Valentin, Stephanie	Elliott, James	Australia, USA	2015
[37]	*Age and Side-Related Morphometric MRI Evaluation of Trunk Muscles in People without Back Pain.*			
	10.1016/j.math.2014.07.007			
	Valentin, Stephanie	Elliott, James	Australia, USA	2015
[67]	*Three-dimensional spatial distribution of lumbar paraspinal intramuscular fat revealed by spatial parametric mapping*			
	10.1007/s00586-024–08559-1			
	Weber, K. A. II	De Leener, B.	Canada	2024
[2]	*Quantifying Lumbar Paraspinal Intramuscular Fat: Accuracy and Reliability of Automated Thresholding Models.*			
	10.1016/j.xnsj.2024.100313			
	Wesselink, E. O.	Weber II, K. A.	USA	2024
[41]	*Preoperative Deltoid Size and Fatty Infiltration of the Deltoid and Rotator Cuff Correlate to Outcomes after Reverse Total Shoulder Arthroplasty.*			
	10.1007/s11999-014–4047-2			
	Wiater, Brett P.	Wiater, J. Michael	USA	2015
[39]	*A Valid and Precise Semiautomated Method for Quantifying Intermuscular Fat Intramuscular Fat in Lower Leg Magnetic Resonance Images.*			
	10.1016/j.jocd.2018.09.007			
	Wong, Andy K. O.	Cheung, Angela M.	Canada	2020
[74]	*Holistic Segmentation of Intermuscular Adipose Tissues on Thigh MRI.*			
	10.1007/978–3-319–66182-7_84			
	Yao, Jianhua	Summers, Ronald M.	USA	2017
[51]	*Effects of Post-Fracture Non-Weight-Bearing Immobilization on Muscle Atrophy, Intramuscular and Intermuscular Adipose Tissues in the Thigh and Calf.*			
	10.1007/s00256-018–2985-6			
	Yoshiko, Akito	Akima, Hiroshi	Japan	2018
[31]	*Measurement of Intramuscular Fat by Muscle Echo Intensity.*			
	10.1002/mus.24656			
	Young, Hui-Ju	Mccully, Kevin K.	USA	2015
[32]	*Correlation of the Features of the Lumbar Multifidus Muscle With Facet Joint Osteoarthritis.*			
	10.3928/01477447-20170531-05			
	Yu, Bo	Liu, Zude	China	2017

**Table A2:** Study characteristics of the eligible studies. Columns specify the anatomical region examined, muscles segmented, type of adipose tissue assessed (Ass. Adip. Tissue), species studied, and cohort size. Differences in anatomical focus and adipose tissue definition across studies are indicated

Ref.	Bodypart Examined	Muscles Segmented	Ass. Adip. Tissue	Patient Species	Cohort Size
[52]	Leg, Thigh	Biceps Femoris, Vastus Lateralis	IMF	human	30
[53]	Leg, Thigh	Adductor Longus, Adductor Magnus, Biceps Femoris-Long Head, Gracilis, Rectus Femoris, Sartorius, Semimembranosus, Semitendinosus, Vastus Intermedius, Vastus Lateralis, Vastus Medialis	IMF	human	30
[76]	Leg, Thigh, Calf	Combined Mask for all visible Muscle Tissue	IMF+IMAT	human	17
[47]	Lumbar Spine	Erector Spinae, Multifidus	unclear	human	37
[77]	Lumbar Spine	Erector Spinae + Multifidus	IMF, IMAT	human	100
[55]	Lumbar Spine	Erector Spinae, Multifidus	IMF, IMF+IMAT	human	37
[69]	Leg, Thigh	Biceps Femoris-Long Head, Biceps Femoris-Short Head, Rectus Femoris, Semimembranosus, Semitendinosus, Vastus Intermedius, Vastus Lateralis, Vastus Medialis	IMF	human	20
[34]	Thoracic Spine	Longissimus Dorsi + Iliocostales, Multifidus, Multifidus + Longissimus Dorsi + Iliocostales	unclear	animal, dachshund, dog	64
[35]	Thoracic Spine	Longissimus Dorsi + Iliocostales, Multifidus, Multifidus + Longissimus Dorsi + Iliocostales	IMF	animal, dog, large breed dog	30
[68]	Leg, Thigh	Combined Mask for all visible Muscle Tissue	IMF+IMAT	human	11
[79]	Leg, Thigh	Combined Mask for all visible Muscle Tissue	IMF	human	11
[60]	Lumbar Spine	Multifidus	IMF	human	45
[26]	Lumbar Spine	Erector Spinae + Multifidus	IMF	human	107
[72]	Shoulder	Deltoid, Infraspinatus, Subscapularis, Supraspinatus, Teres Minor, Trapezius	IMF	human	7
[73]	Pelvis	Gluteus Maximus, Gluteus Medius/Minimus	IMF, IMF+IMAT	human	19
[71]	Shoulder	Supraspinatus	IMF	human	93
[58]	Leg	Combined Mask for all visible Muscle Tissue	IMF+IMAT	human	26
[57]	Leg	Combined Mask for all visible Muscle Tissue	IMF+IMAT	human	26
[16]	Lumbar Spine	Erector Spinae, Multifidus	IMF	human	75
[22]	Cervical Spine	Multifidus + Semispinalis Cervicis, Semispinalis Capitis, Splenius Capitis	IMF	human	20
[15]	Lumbar Spine	Erector Spinae, Multifidus	unclear	human	99
[17]	Lumbar Spine	Erector Spinae, Multifidus	IMF+IMAT	human	33
[13]	Cervical Spine	Multifidus, Semispinalis Capitis, Semispinalis Cervicis, Splenius Capitis	IMF	human	38
[43]	Lumbar Spine	Erector Spinae, Multifidus	unclear	human	30
[12]	Lumbar Spine	Erector Spinae, Multifidus, Psoas Major	IMF+IMAT	human	36
[10]	Cervical Spine	Multifidus, Multifidus + Semispinalis Cervicis, Semispinalis Capitis, Semispinalis Cervicis, Splenius Capitis	IMF	human	10
[54]	Lumbar Spine	Erector Spinae, Multifidus	IMF	human	28
[9]	Lumbar Spine	Erector Spinae + Multifidus	IMF+IMAT	human	47
[24]	Lumbar Spine	Erector Spinae + Multifidus, Psoas Major	IMF	human	55
[29]	Lumbar Spine	Erector Spinae, Multifidus, Psoas Major, Quadratus Lumborum	IMF	human	160
[20]	Lumbar Spine	Erector Spinae, Multifidus, Psoas Major, Quadratus Lumborum	IMF	human	262
[66]	Leg, Thigh, Calf	Combined Mask for all visible Muscle Tissue	IMAT	human	57
[56]	Lumbar Spine	Multifidus	IMF	human	70
[40]	Shoulder	Supraspinatus, supraspinatus fossa	unclear	human	38
[21]	Lumbar Spine	Erector Spinae, Multifidus	IMF+IMAT	human	62
[27]	Lumbar Spine	Erector Spinae + Multifidus	IMF+IMAT	human	35
[14]	Lumbar Spine	Erector Spinae, Multifidus	IMF+IMAT	human	60
[18]	Lumbar Spine	Erector Spinae + Multifidus, Psoas Major	IMF+IMAT	human	41
[19]	Lumbar Spine	Erector Spinae + Multifidus	IMF+IMAT	human	18
[11]	Lumbar Spine	Erector Spinae, Multifidus, Psoas Major	IMF+IMAT	human	35
[25]	Lumbar Spine	Erector Spinae + Multifidus	unclear	human	10
[28]	Lumbar Spine	Erector Spinae, Multifidus, Psoas Major	unclear	human	28
[59]	Lumbar Spine	Erector Spinae + Multifidus, Psoas Major	IMF	human	95
[44]	Lumbar Spine	Erector Spinae, Multifidus, Psoas Major, Quadratus Lumborum	IMF+IMAT	human	60
[49]	Leg	Combined Mask for all visible Muscle Tissue	IMF+IMAT	human	10
[50]	Leg, Thigh	Adductor Magnus, Biceps Femoris-Long Head, Vastus Lateralis	IMF	human	32
[33]	Lumbar Spine	Erector Spinae, Multifidus, Psoas Major	IMF	human	73
[70]	Leg, Thigh	Combined Mask for all visible Muscle Tissue	IMAT	human	13
[48]	Lumbar Spine	Erector Spinae, Multifidus, Psoas Major, Quadratus Lumborum	IMF	human	11
[23]	Lumbar Spine	Erector Spinae, Multifidus, Psoas Major, Quadratus Lumborum	IMF	human	26
[65]	Lumbar Spine	Erector Spinae, Multifidus, Psoas Major	IMF	human	796
[45]	Lumbar Spine	Erector Spinae, Multifidus, Psoas Major	unclear	human	120
[38]	Lumbar Spine	Erector Spinae, Erector Spinae + Multifidus, Multifidus, Psoas Major, Quadratus Lumborum	IMF	human	13
[42]	Lumbar Spine	Erector Spinae, Multifidus, Psoas Major	IMF	human	80
[46]	Lumbar Spine	Erector Spinae, Multifidus	IMF	human	80
[36]	Lumbar Spine	Erector Spinae, Multifidus, Psoas Major	IMF	animal, human, sheep	41
[37]	Abdomen, Lumbar Spine	Erector Spinae, Multifidus, Psoas Major, Rectus Abdominis	IMF	human	24
[67]	Lumbar Spine	Erector Spinae, Multifidus, Psoas Major	IMF	human	76
[2]	Lumbar Spine	Erector Spinae, Multifidus, Psoas Major	IMF	human	30
[41]	Shoulder	Deltoid, Infraspinatus, Subscapularis, Supraspinatus, Teres Minor	IMF	human	30
[39]	Leg, Calf	Combined Mask for all visible Muscle Tissue	IMF+IMAT	human	110
[74]	Leg, Thigh	Combined Mask for all visible Muscle Tissue	IMF, IMAT	human	104
[51]	Leg, Thigh, Calf	Biceps Femoris Short Head + Biceps Femoris Long Head + Semitendinosus + Semimembranosus, Peroneus, Rectus Femoris + Vastus Lateralis + Vastus intermedius + Vastus Medialis, Sartorius + Gracilis + Adductor Longus + Adductor Magnus, Soleus + Medial Gastrocnemius + Lateral Gastrocnemius, Tibial Anterior + Extensor Digitorum Longus + Extensor Halluces, Tibial Posterior + Flexor Hallucis Longus + Flexor Digitorum Longus	IMF, IMF+IMAT	human	8
[31]	Leg, Thigh, Calf	Biceps Femoris, Medial Gastrocnemius, Rectus Femoris, Tibialis Anterior	IMF	human	31
[32]	Lumbar Spine	Multifidus	IMF	human	160

**Table A3:** Overview of MRI parameters and methodological details in the surveyed studies. The table summarizes acquisition and analysis parameters used for muscle fat assessment. “MRI parameters” specifies the reported image acquisition parameters from conventional MRI protocols. “Image preproc. Method” abbreviates “image preprocessing method” and lists any applied image enhancement or correction algorithms. “Muscle Segmentation Method” and “Fat Quantification Method” describe the approaches and software used for muscle segmentation and fat quantification, respectively

Ref.	MRI parameters		
	Image Preproc. Method	Muscle Segmentation Method	Fat Quantification Method
[52]	3 Tesla, MAGNETOM Verio - Siemens, Spin-Echo, T1w, TE 11 ms, TR 604 ms, body coil, field of view 256 × 256 mm, interslice gap 0 mm, slice thickness 10 mm, voxel resolution 0.75 mm		
	N3 Method	Manual, Medical Image Processing Analysis and Visualization software (MIPAV)	MIPAV, Otsu on 6 25 mm*25 mm ROIs − 3 in Vastus Intermedialis (VI) and 3 in subcutaneous fat
[53]	3 Tesla, MAGNETOM Verio - Siemens, Spin-Echo, T1w, TE 11 ms, TR 604 ms, body coil, field of view 256 × 256 mm, interslice gap 0 mm, slice thickness 10 mm, voxel resolution 0.75 mm		
	N3 Method	Manual, Medical Image Processing Analysis and Visualization software (MIPAV)	MIPAV, Otsu on 6 25 mm*25 mm ROIs − 3 in Vastus Intermedialis (VI) and 3 in subcutaneous fat
[76]	3 Tesla, MAGNETOM Verio - Siemens, Spin-Echo, T1w, TE 11 ms, TR 604 ms, body coil, field of view 256 × 256 mm, interslice gap 0 mm, slice thickness 10 mm, voxel resolution 0.75 mm		
	N3 Method	Manual, Medical Image Processing Analysis and Visualization software (MIPAV)	MIPAV, Otsu on 6 25 mm*25 mm ROIs − 3 in Vastus Intermedialis (VI) and 3 in subcutaneous fat
[47]	1.5 Tesla, Philips Achieva, T2w, TE 100 ms, TR 3332.29 ms, matrix size 168x121, reconstructed pixel spacing 0.47x0.47 mm, slice thickness 4 mm		
		Manual, OsiriX (Plugin)	Otsu, If FI larger than 50% Yen’s algorithm, OsiriX (Plugin)
[77]	Aera Siemens − 1.5 Tesla, Other Parameters are differing, Siemens Trio − 3 Tesla, Siemens or Philips Ingenia − 3 Tesla, T2w		
		U-Net, Python	U-Net, Python
[55]	T2w		
		Manual	Intercept of Curves = Threshold, histogram of ROI, two-term Gaussian model, Matlab
[69]	3 Tesla, 32-element cardiac coil, Achieva TX Philips, T1w, TE 12 ms, TR 700 ms, Turbo Spin Echo, field of view 180x180mm, matrix size 256x188mm, slice thickness 4 mm		
	N3 Method	Either in T1w or on mDixon and then resampled to resolution of T1w, Manual, Masks Eroded by 2 mm to avoid inclusion of extramuscular tissue	Fuzzy C-Means Clustering, (exponent 2 - maxIt 200 - tol 0.01), Matlab
[34]	1.5 Tesla, Philips Intera, T1w, TE 8-120 ms, TR 400–3680.79 ms, interslice gap 2.8–4.4 mm, slice thickness 2.5-4 mm		
		Manual, OsiriX	Mean Signal Intensity divided by subcutaneous fat ROI signal intensity, Osirix
[35]	1.5 Tesla, Philips Intera, T1w, TE 8–11 ms, TR 484–1151 ms, interslice gap 3.3–3.85 mm, matrix size 288 × 288- 400x400, slice thickness 3–3.5 mm		
		Manual, OsiriX	Mean Signal Intensity divided by subcutaneous fat ROI signal intensity, Osirix
[68]	3 Tesla, Echo Train Length 3, Fast Spin-Echo (FSE), Philips, T1w, TE 20 ms, TR 500 ms, field of view 330 × 330 mm, matrix size 784x784		
		3D Livewire, Matlab	Fuzzy C-Means Clustering (4 classes), Matlab
[79]	T1w		
		1: Pseudo-Label Generation: MC-Net, 2: Pseudo-Label Correction: Inspired by source-free domain adaptation approaches, 3: Noise Robust Loss, 4: Augmentation, Python	1: Pseudo-Label Generation: MC-Net, 2: Pseudo-Label Correction: Inspired by source-free domain adaptation approaches, 3: Noise Robust Loss, 4: Augmentation, Python
[60]	T1w, T2w		
		Manual, SliceOmatic	Histogram crossing 0, SliceOmatic
[26]	T1w		
		Manual, Matlab	ROI in subcutaneous fat, Matlab
[72]	3 Tesla, T1w		
		Manual, Medical Image Processing Analysis and Visualization software (MIPAV)	Fuzzy C-Means Clustering, (number of desired classes: 2 - desired exponent value: 2 - end tolerance: 0.01 - maximum number of iteration: 200 - signal threshold: −1.024 - region: VOI region - segmentation: hard and fuzzy both), MIPAV
[73]	3 Tesla, Magnetom Prisma Siemens, Magnetom Trio Siemens, T1w, Turbo Spin Echo		
		Manual, Medical Image Processing Analysis and Visualization software (MIPAV)	Fuzzy C-Means Clustering, (Single Channel - Number of desired classes: 2 - desired exponent value: 2 - end tolerance: 0.01 - maxIt: 200 - signal threshold: 0.0- region: VOI region - segmentation: hard and fuzzy both), MIPAV
[71]	T1w		
		Manual, Medical Image Processing Analysis and Visualization software (MIPAV)	Fuzzy C-Means Clustering, (number of desired classes: 2 - desired exponent value: 2 - end tolerance: 0.01 - maximum number of iteration: 200 - signal threshold: 0.0 - region: VOI region - segmentation: hard and fuzzy both), MIPAV
[58]	1.5 Tesla, General Electric (GE) Signa HDxt, Spin-Echo, T1w, TE 14 ms, TR 540 ms		
		CSOIsoGenerator (Contour Segmentation Objects), MeVisLab	Multigaussian fit, MeVisLab
[57]	1.5 Tesla, General Electric (GE) Signa HDxt, Spin-Echo, T1w, TE 14 ms, TR 540 ms		
		CSOIsoGenerator (Contour Segmentation Objects, MeVisLab	Multigaussian fit, MeVisLab
[16]	1.5 Tesla, Magnetom SP 4000 Siemens, T2w		
		ImageJ, Manual	4–6 ROIs from lean muscle - highest value is threshold, ImageJ
[22]	T2w		
		ImageJ, Manual	4–6 ROIs from lean muscle - highest value is threshold, ImageJ
[15]	1.5 Tesla, Magnetom SP 4000 Siemens, T2w		
		ImageJ, Manual	4–6 ROIs from lean muscle - highest value is threshold, ImageJ
[17]	T2w		
		ImageJ, Manual	4–6 ROIs from lean muscle - highest value is threshold, ImageJ
[13]	T2w		
		Manual, OsiriX	4–6 ROIs from lean muscle - highest value is threshold, OsiriX
[43]	1.5 Tesla, Siemens Avanto, T2w, TE 113 ms, TR 4000 ms, Turbo Spin Echo		
	CLAHE Algorithm	Manual	Otsu, Matlab
[12]	T2w		
		ImageJ, Manual	4–6 ROIs from lean muscle - highest value is threshold, ImageJ
[10]	T2w		
		ImageJ, Manual	4–6 ROIs from lean muscle - highest value is threshold, ImageJ
[54]	3 Tesla, T1w, TE 2.3 ms, TIM Trio Siemens, TR 4 ms		
		MITK, Manual	2 Intensity Peaks - midpoint of peaks is threshold, Matlab
[9]	1.5 Tesla, General Electric, T2w, interslice gap 1 mm, slice thickness 4 mm		
		ImageJ, Manual	4–6 ROIs from lean muscle - highest value is threshold, ImageJ
[24]	T2w		
		ImageJ, Manual	4–6 ROIs from lean muscle - highest value is threshold, ImageJ
[29]	3 Tesla, Discovery 750 GE, T1w		
		ImageJ, Manual	Constant value of 120, ImageJ
[20]	T2w		
		ImageJ, Manual	4–6 ROIs from lean muscle - highest value is threshold, ImageJ
[66]	1.5 Tesla, Siemens Avanto, T1w, TE 11 ms, TR 578 ms, field of view 400x400mm, interslice gap 2.8–4.4 mm, matrix size 160x320, slice thickness 4 mm		
		Fuzzy C-Means - (Background+Bone+Vessel - adipose tissue - muscle tissue), Morphologic closing- keeping muscle compartment, Polygonal Active Contour Algorithm (Snake)	Fuzzy C-Means Clustering
[56]	3 Tesla, MAGNETOM Verio - Siemens, T1w, field of view 180 mm2, matrix size 256x256, slice thickness 4 mm		
		Manual	2 Intensity Peaks - midpoint of peaks is threshold, Matlab
[40]	3 Tesla, T1w, intra-articular injection 12 ml gadodiamide concentration 2 mmol/ L		
		Manual	Matlab, Otsu
[21]	T2w		
		ImageJ, Manual	4–6 ROIs from lean muscle - highest value is threshold, ImageJ
[27]	1.5 Tesla, Philips Achieva, T2w, field of view 180 mm2, interslice gap 1 mm, slice thickness 4 mm		
		ImageJ, Manual	Constant value of 120, ImageJ
[14]	1.5 Tesla, Siemens Avanto, T2w, TE 113 ms, TR 4000 ms, Turbo Spin Echo, slice thickness 3 mm		
		Amira, ImageJ, Manual	4–6 ROIs from lean muscle - highest value is threshold, Overlapping Histogram of Muscle and Subcutaneous fat region, Amira, ImageJ
[18]	1.5 Tesla, 23-Na imaging, 3 Tesla, Siemens Avanto, Siemens Skyra, T1w, TE 12/11 ms, TR 600/630 ms, echo train length 4/2, field of view 280/200 mm, matrix size 384x384, slice thickness 5 mm		
		ImageJ, Manual	4–6 ROIs from lean muscle - highest value is threshold, ImageJ
[19]	3 Tesla, Siemens Prisma, T2w		
		ImageJ, Manual	4–6 ROIs from lean muscle - highest value is threshold, ImageJ
[11]	3 Tesla, General Electric, T2w, TE 98 ms, TR 3800 ms, field of view 180 mm2, matrix size 512x512, slice thickness 4 mm		
		ImageJ, Manual	4–6 ROIs from lean muscle - highest value is threshold, ImageJ
[25]	3 Tesla, T1w, TE 9.5 ms, TR 500 ms, slice thickness 4 mm		
		Manual, Matlab	ROI in subcutaneous fat, Matlab
[28]	1.5 Tesla, Philips Achieva, T2w		
		ImageJ, Manual	Constant value of 120, ImageJ
[59]	T2w		
		ITK-Snap, Manual	Threshold is mean + standard deviation of ROI, Matlab
[44]	3 Tesla, Siemens Skyra, T2w		
		U-Net, Python	Otsu, Matlab
[49]	T1w		
		Manual, Medical Image Processing Analysis and Visualization software (MIPAV)	Otsu on 6 25 mm*25 mm ROIs − 3 in Vastus Intermedialis (VI) and 3 in subcutaneous fat, ROI of 50% subcutaneous fat and 50% muscle - Midpoint is threshold, MIPAV
[50]	3 Tesla, MAGNETOM Verio - Siemens, Spin-Echo, T1w, TE 12 ms, TR 604 ms, field of view 256 × 256 mm, interslice gap 0 mm, slice thickness 10 mm		
	N3 Method	Manual, medical Image Processing Analysis and Visualization software (MIPAV)	Otsu on 6 25 mm*25 mm ROIs − 3 in Vastus Intermedialis (VI) and 3 in subcutaneous fat, MIPAV
[33]	3 Tesla, Discovery MR750w, Magnetom Prisma Siemens, T2w, TE 89–100 ms, TR 3200–4000 ms		
		ImageJ, Manual	ImageJ, Mean signal intensity divided by subcutaneous fat ROI signal intensity
[70]	1.5 Tesla, Echo Train Length 3, Philips Achieva, T1w, TE 15 ms, TR 550 ms, interslice gap 0 mm, slice thickness 6 mm		
	local entropy minimization with a bicubic spline model (LEMS)	Fuzzy C-Means Clustering, Morphological opening and closing, Matlab	Matlab, Fuzzy C-Means Clustering
[48]	3 Tesla, Fast Spin-Echo (FSE), GE MR 750, T1w, T2w, TE 13 ms, TE 56 ms, TR 594 ms, TR 8414 ms, field of view 180x180mm, interslice gap 4 mm, slice thickness 4 mm, voxel resolution 0.35 mm		
	CLAHE Algorithm	2D V-Net, Python	Gaussian curve fitting, Otsu, Quadratic Discriminant Analysis, k-means clustering, Python
[23]	1.5 Tesla, 3 Tesla, T2w, TE 84–125 ms, TR 3633.3–7500 ms, Turbo Spin Echo, field of view 180 × 144- 200 × 200 mm, matrix size 320–512 ×185–256 mm		
		ImageJ, Manual	4–6 ROIs from lean muscle - highest value is threshold, ImageJ
[65]	1.5 Tesla, Fast Spin-Echo (FSE), Philips Achieva, T2w, TE 100 ms, TR 2100 ms, field of view 180x180mm		
		Manual	K-means Clustering
[45]	3 Tesla, Philips, T2w, TE 120 ms, TR 3140 ms, Turbo Spin Echo, echo train length 17, field of view 240 mm, interslice gap 0.4 mm, matrix size 400x400, slice thickness 4 mm		
		Mask R-CNN, Python	Otsu, Python
[38]	1.5 Tesla, Siemens, Spin-Echo, T1w, TE 13 ms, TR 879 ms, field of view 230 × 230 mm, flexible spine coil, interslice gap 1.5 mm, matrix size 480x640, slice thickness 5 mm		
		ImageJ, Manual	ImageJ, Mean signal intensity divided by visceral fat ROI signal intensity
[42]	1.5 Tesla, T2w		
		Manual, OsiriX	Otsu, OsiriX
[46]	1.5 Tesla, T2w		
		Manual, OsiriX	Otsu, OsiriX
[36]	1.5 Tesla, Other Parameters are differing, T1w		
		Analyze, Manual	Mean signal intensity divided by isceral fat ROI signal intensity, Analyze
[37]	1.5 Tesla, Philips Achieva, T1w, TE 4.6 ms, TR 9.3 ms, field of view 345 × 345 mm, interslice gap 1 mm, slice thickness 10 mm		
		Analyze, Manual	Mean signal intensity divided by isceral fat ROI signal intensity, Analyze
[67]	T2w,TE 116 ms,TR 5000 ms,slice thickness 4 mm, 3 Tesla		
		Manual	Gaussian Mixture Modelling − 2 Classes, Python
[2]	3 Tesla, Magnetom Vida Siemens, T2w, TE 9.4 ms, TR 3.3 ms, matrix size 320x320, slice thickness 3 mm, voxel resolution 0.59x0.59 mm		
		Manual	Gaussian Mixture Modelling − 2 Classes, Gaussian Mixture Modelling − 3 Classes, K-Means Clustering- 2 classes, K-Means Clustering 3 Classes, Python
[41]	3 Tesla, T1w		
		Manual	Adaptive Thresholding based on local variances in signal intensity, Matlab
[39]	T1w, T2w		
		Manual	Iterative Threshold Optimization, Matlab
[74]	T1w, TE 20 ms, TR 600 ms, slice thickness 8 mm, voxel resolution 0.98 mm		
		Threshold + Morphological opening and closing + Holistically-nested Edge Detection (HED) and Dual Active Contour Model for fascia lata segmentation	Holistic Region Neural Network (based on finetuned VGG-16) and Threshold of 0.5, Caffe library (either Matlab or Python)
[51]	1.5 Tesla, Excel ART Toshiba, Spin-Echo, T1w, TE 15 ms, TR 625 ms, field of view 250 × 250 mm, interslice gap 0 mm, matrix size 512x512, slice thickness 10 mm		
	N3 Method	Manual, Medical Image Processing Analysis and Visualization software (MIPAV)	MIPAV, Otsu on 6 25 mm*25 mm ROIs − 3 in Vastus Intermedialis (VI) and 3 in subcutaneous fat
[31]	3 Tesla, General Electric, T1w, TE min full, TR 800 ms, matrix size 1024x1024		
		ImageJ, Manual	ROIs of fat and muscle - calculation of a fat percentage - calculation of weighted fat and muscle voxel counts, ImageJ
[32]	3 Tesla, Fast Spin-Echo (FSE), Siemens, T2w, TE 120 ms, TR 3220 ms, field of view 310 × 310 mm, matrix size 512x512, slice thickness 4 mm		
		ImageJ, Manual	ImageJ, Mean signal intensity divided by subcutaneous fat ROI signal intensity

**Table A4:** Information on software-related aspects for the surveyed studies. The table summarizes methodological details regarding software and implementation. “SW Avail.” abbreviates “software availability” and indicates whether the software used for image analysis was freely available or commercial; “Code Avail.” abbreviates “code availability” and specifies the availability of source code. The “Fat Quantification Subcategory” column denotes the category of methodological approach used for fat quantification in conventional MRI, while “Autom. Degree” reflects the level of automation of the analysis pipeline. “# QM tested” abbreviates “number of quantification methods tested”, and “Valid. Ref. Standard” abbreviates “validation reference standard” and lists the reference standard applied for validation

Ref.	SW Avail.	Code Avail.	Fat Quantification Subcategory	Autom. Degree	# QM tested	Valid. Ref. Standard
[52]	free		Histogram Shape-Based	2	1	MRS
[53]	free		Histogram Shape-Based	2	1	
[76]		no	Supervised Machine Learning	4*	1	
[47]	commercial		Histogram Shape-Based	4*	1	2pt Dixon (fat image only)
[77]	free	no	Supervised Machine Learning	4	1	
[55]	commercial	no	Histogram Shape-Based	4*	1	
[69]	commercial	no	Unsupervised Machine Learning	4*	1	2pt Dixon
[34]	commercial		Local Signal Intensity-Based	2	1	
[35]	commercial		Local Signal Intensity-Based	2	1	
[68]	commercial	no	Unsupervised Machine Learning	4*	1	
[79]	free	no	Supervised Machine Learning	4*	1	
[60]	commercial		Histogram Shape-Based	2	1	
[26]	commercial	no	Local Signal Intensity-Based	2	1	
[72]	free		Unsupervised Machine Learning	4*	1	6pt Dixon
[73]	free		Unsupervised Machine Learning	4*	1	
[71]	free		Unsupervised Machine Learning	4*	1	
[58]	free		Histogram Shape-Based	4*	1	
[57]	free		Histogram Shape-Based	4*	1	
[16]	free		Local Signal Intensity-Based	2	1	
[22]	free		Local Signal Intensity-Based	2	1	
[15]	free		Local Signal Intensity-Based	2	1	
[17]	free		Local Signal Intensity-Based	2	1	
[13]	commercial		Local Signal Intensity-Based	2	1	
[43]	commercial	yes	Histogram Shape-Based	4*	1	
[12]	free		Local Signal Intensity-Based	2	1	
[10]	free		Local Signal Intensity-Based	2	1	
[54]	commercial	no	Histogram Shape-Based	4*	1	
[9]	free		Local Signal Intensity-Based	2	1	
[24]	free		Local Signal Intensity-Based	2	1	
[29]	free		Local Signal Intensity-Based	3	1	
[20]	free		Local Signal Intensity-Based	2	1	
[66]		no	Unsupervised Machine Learning	4*	1	
[56]	commercial	no	Histogram Shape-Based	4*	1	
[40]	commercial	no	Histogram Shape-Based	4*	1	MRS
[21]	free		Local Signal Intensity-Based	2	1	
[27]	free		Local Signal Intensity-Based	3	1	
[14]	commercial, free		Histogram Shape-Based, Local Signal Intensity-Based	2	2	
[18]	free		Local Signal Intensity-Based	2	1	
[19]	free		Local Signal Intensity-Based	2	1	
[11]	free		Local Signal Intensity-Based	2	1	2pt Dixon
[25]	commercial	no	Local Signal Intensity-Based	2	1	
[28]	free		Local Signal Intensity-Based	3	1	
[59]	commercial	no	Histogram Shape-Based	4*	1	
[44]	commercial	no	Histogram Shape-Based	4*	1	
[49]	free		Histogram Shape-Based	2, 4*	2	
[50]	free		Histogram Shape-Based	2	1	2pt Dixon
[33]	free		Local Signal Intensity-Based	2	1	
[70]	commercial		Unsupervised Machine Learning	4*	1	
[48]	free	no	Unsupervised Machine Learning, Histogram Shape-Based, Supervised Machine Learning	4*	4	6pt Dixon
[23]	free		Local Signal Intensity-Based	2	1	
[65]		no	Unsupervised Machine Learning	4*	1	
[45]	free	no	Histogram Shape-Based	4*	1	
[38]	free		Local Signal Intensity-Based	2	1	
[42]	commercial		Histogram Shape-Based	4*	1	
[46]	commercial		Histogram Shape-Based	4*	1	
[36]	commercial		Local Signal Intensity-Based	2	1	
[37]	commercial		Local Signal Intensity-Based	2	1	
[67]	free	yes	Unsupervised Machine Learning	4*	1	
[2]	free	yes	Unsupervised Machine Learning, Histogram Shape-Based	4*	4	2pt Dixon
[41]	commercial	no	Histogram Shape-Based	4*	1	
[39]	commercial	no	Local Signal Intensity-Based	4*	1	
[74]	commercial or free	no	Supervised Machine Learning	4*	1	
[51]	free		Histogram Shape-Based	2	1	
[31]	free		Local Signal Intensity-Based	2	1	
[32]	free		Local Signal Intensity-Based	2	1	

## Abbreviations

AM: Adductor magnus
BF: Biceps femoris
BF-L: Biceps femoris long head
BF-S: Biceps femoris short head
CNN: Convolutional neural network
CSE-MRI: Chemical shift encoded magnetic resonance imaging
D: Deltoid
EMC: Echo modulation curve
EMCL: Extramyocellular lipids
ES: Erector spinae
FCM: Fuzzy c-means
FF: Fat fraction
FI: Fatty infiltration
GMM: Gaussian Mixture Modelling
ICC: Intraclass correlation coefficient
IMAT: Intermuscular adipose tissue
IMCL: Intramyocellular lipids
IMF: Intramuscular fat
IS: Infraspinatus
MF: Multifidus
MRI: Magnetic resonance imaging
MRS: Magnetic resonance spectroscopy
PICO: Population Intervention Comparison Outcome
PM: Psoas major
PRISMA: Preferred Reporting Items for Systematic reviews and Meta-Analyses
QDA: Quadratic discriminant analysis
QL: Quadratus lumborum
qMRI: Quantitative magnetic resonance imaging
RF: Rectus femoris
ROI: Region of interest
SC: Subscapularis
SF: Supraspinatus fossa
SM: Semimembranosus
SS: Supraspinatus
ST: Semitendinosus
T: Trapezius
TM: Teres minor
VI: Vastus intermedius
VL: Vastus lateralis
VM: Vastus medialis
